# Modulating Stress Proteins in Response to Therapeutic Interventions for Parkinson’s Disease

**DOI:** 10.3390/ijms242216233

**Published:** 2023-11-12

**Authors:** Serena Silvestro, Ivana Raffaele, Emanuela Mazzon

**Affiliations:** IRCCS Centro Neurolesi Bonino Pulejo, Via Provinciale Palermo, Contrada Casazza, 98124 Messina, Italy; serena.silvestro@irccsme.it (S.S.); ivana.raffaele@irccsme.it (I.R.)

**Keywords:** heat shock proteins, Parkinson’s disease, neuroregeneration, stress protein modulation, protein misfolding, pharmacological modulation, nonpharmacological interventions

## Abstract

Parkinson’s disease (PD) is a neurodegenerative illness characterized by the degeneration of dopaminergic neurons in the substantia nigra, resulting in motor symptoms and without debilitating motors. A hallmark of this condition is the accumulation of misfolded proteins, a phenomenon that drives disease progression. In this regard, heat shock proteins (HSPs) play a central role in the cellular response to stress, shielding cells from damage induced by protein aggregates and oxidative stress. As a result, researchers have become increasingly interested in modulating these proteins through pharmacological and non-pharmacological therapeutic interventions. This review aims to provide an overview of the preclinical experiments performed over the last decade in this research field. Specifically, it focuses on preclinical studies that center on the modulation of stress proteins for the treatment potential of PD. The findings display promise in targeting HSPs to ameliorate PD outcomes. Despite the complexity of HSPs and their co-chaperones, proteins such as HSP70, HSP27, HSP90, and glucose-regulated protein-78 (GRP78) may be efficacious in slowing or preventing disease progression. Nevertheless, clinical validation is essential to confirm the safety and effectiveness of these preclinical approaches.

## 1. Introduction

Parkinson’s disease (PD) is a neurodegenerative disorder characterized by the loss of dopaminergic neurons in the substantia nigra of the brain. This condition affects millions of people worldwide, and its prevalence continues to increase as the global population ages [1,2]. Parkinson’s is associated with a range of motor symptoms, including tremors, stiffness, bradykinesia, and postural instability, as well as psychological symptoms, such as depression, anxiety, sleep disturbances, and cognitive difficulties [3].

The pathogenesis of PD is complex and multifactorial and involves several molecular and cellular mechanisms. Although the precise nature of the disease’s pathological mechanisms remains enigmatic, several factors have been implicated, including mitochondrial dysfunction, oxidative stress, calcium dysregulation, inflammation, and protein turnover [4]. Notably, misfolded protein accumulation and cellular stress play a pivotal role in the progressive degeneration of dopaminergic neurons in the substantia nigra. Alpha-synuclein (α-syn), a protein involved in synaptic vesicle regulation, is responsible for the formation of protein aggregates known as Lewy bodies, which are observed in the surviving neurons in the brain of PD patients [5]. Whereas the majority of PD cases are sporadic, it is suspected that genetic and environmental factors contribute to its etiology [6]. Hereditary PD has been linked to over twenty genes [7]. Notable examples include *SNCA* (*PARK1*, *PARK4*), parkin (*PARK2*), DJ-1 (PARK7), LRRK2 (*PARK8*) *UCHL1* and *PINK1*, which respectively encode α-syn, parkin (PARK), protein/nucleic acid deglycase DJ-1, leucine-rich repeat kinase 2 (LRRK2), ubiquitin C-terminal hydrolase L1 (UCHL1), and phosphatase and tensin homolog-induced kinase 1 (PINK1) [8,9,10,11]. In familial PD, these various gene mutations suggest that alteration in protein conformation and/or degradation may contribute to the disease.

In PD, there appears to be a compromised ability for stress proteins to counteract the accumulation of misfolded proteins and prevent cell damage. Protein chaperones and co-chaperones play a crucial role in assisting other proteins to fold correctly and achieve a functional state. These chaperones and co-chaperones interact with PD-related proteins [12]. Research on chaperones and PD has primarily relied on cell and animal models, with limited investigations on the human brain. Consequently, understanding the relationship between chaperones and PD in humans remains challenging. Identifying and comprehending the mechanisms of this condition is relevant for exploring new potential strategies to mitigate PD progression. Such strategies may include chaperone-inducing drugs, proteasome inhibitors, antioxidant treatments, gene therapies, and cell therapies [13]. 

In this review, we will specifically focus on studies from the last decade that have assessed the modulation of stress proteins through therapeutic interventions in PD. Our aim is to provide a detailed overview of the preclinical data in this research area. Additionally, we will analyze both preclinical and clinical evidence to evaluate whether these therapeutic approaches effectively alleviate PD symptoms and modulate stress proteins. 

## 2. Methods

This review was conducted by searching the scientific literature using databases such as PubMed. Specifically, to compile the sections “4. Therapeutic Approaches for Modulating Stress Proteins in PD” and “5. Therapeutic compounds as potential treatment in PD: insights from preclinical studies on stress protein modulation”, a bibliographic search was performed using the following keywords: “Parkinson’s disease AND Molecular chaperones AND Therapeutics AND Pre-clinical studies”, or “Parkinson’s disease AND Ubiquitin-proteasome system AND Therapeutics AND Preclinical studies”, or “Molecular chaperones AND Therapeutics AND Preclinical studies”, or “Heat shock protein AND Treatment AND Parkinson’s disease”, or “HSP90 AND Treatment AND Parkinson’s disease”, or “HSP70 AND Treatment AND Parkinson’s disease”, or “HSP27 AND Treatment AND Parkinson’s disease” or “HDAC6 AND Treatment AND Parkinson’s disease”, or “Hsc70-interacting protein (CHIP) AND Parkinson’s disease”, or “Adeno associated virus AND Heat shock protein AND Parkinson’s disease” or “Hsf-1 inducer AND Parkinson’s disease”, or “Parkinson’s disease AND Ubiquitin-proteasome system AND Therapeutics AND Preclinical studies”.

**Figure 1:**
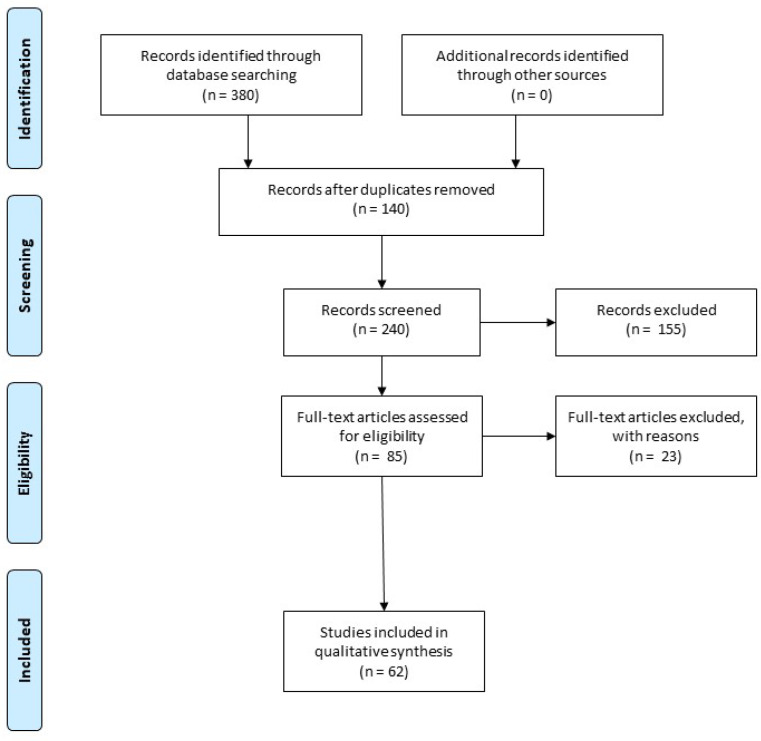
PRISMA flow diagram detailing the methodology that was applied to choose the preclinical studies that were used for the writing of the review. Duplicate articles were excluded from the total number of studies that were recorded. We selected the articles that described therapeutic strategies for improving PD symptoms and modulating stress proteins in preclinical studies. (The PRISMA Statement is published in [14]).

For this review, we focused on studies published in the last decade that centered on the modulation of stress proteins using both pharmacological and non-pharmacological therapeutic interventions within the context of treating PD. We aimed to provide an overview of the in vitro and in vivo evidence, excluding reviews and non-relevant publications, as indicated in the PRISMA flow diagram (Figure 1).

## 3. Heat Shock Proteins (HSPs) in PD: An Overview of Mechanisms and Implications

The excessive accumulation of proteins within dopaminergic neurons can compromise organelle function, leading to cell death and the characteristic neurodegeneration of PD [15]. Under normal conditions, stress proteins, such as heat shock proteins (HSPs) and endoplasmic reticulum (ER)-associated stress proteins, are synthesized by cells in response to different stress-inducing factors. These proteins play an important role in mitigating both the external and internal stressors that could potentially cause molecular damage [16,17]. Consequently, cells have developed a protective “quality control” system known as the stress response to maintain the integrity of proteins within cells, ensuring their proper functioning and correct folding [18]. In more complex organisms such as eukaryotes, there are several families of HSPs and each is classified according to their molecular weight. These families include Hsp40, Hsp60, Hsp70, Hsp90, Hsp100, and the small HSPs [19]. The activity of these HSPs is regulated by another class of proteins called “co-chaperones”, which can be categorized into several groups based on their functional domains, including the BAG domain, the tetratricopeptide domain, and the J domain [20].

Lewy bodies, a hallmark of PD, contain ubiquitinated α-syn and specific HSPs such as HSP70, HSP90, HSP27, HSP40, and HSP110 [21].

Mitochondrial dysfunction is another significant contributor to PD development. A decrease in mitochondrial chaperones has been observed in the substantia nigra pars compacta and serum of PD patients and is potentially related to disease progression [22]. HSP60 is another chaperone involved in the folding within mitochondria [23]. Mitochondrial protein dysfunction can result in excessive oxidative stress and cell damage, processes that are correlated with PD [24].

The evidence of the close links between stress proteins and protein aggregates in PD suggests that the correct functioning of molecular chaperones and protein degradation mechanisms is essential to prevent the formation of pathological aggregates. Notably, the combination of mitochondrial dysfunction, ER stress, and cytosolic dopamine levels collectively contributes to cellular oxidative stress in PD [25]. The accumulation of misfolded proteins and protein aggregates disrupts the redox balance of neuronal cells, leading to an increase in reactive oxygen species (ROS) production [26]. ROS are highly reactive molecules that can cause damage to lipids, proteins, and nucleic acids [27] and may result from protein misfolding, toxic substances, inflammation, or other pathological processes [28]. Thus, the modulation of chaperone proteins, the enhancement of protein degradation mechanisms, and the use of gene therapies for specific mutations may be potential therapeutic strategies to counteract neuronal degeneration and slow down the progression of PD [29].

### 3.1. Protein Misfolding and Aggregation

In PD, the protein α-syn assumes an abnormal three-dimensional conformation due to misfolding, which leads to its aggregation and the formation of Lewy bodies, a hallmark of PD [30]. Consequently, the increased burden of improperly folded proteins may overwhelm the chaperones’ capacity to assist in proper folding, thus promoting aggregate formation [31]. Molecular chaperones play a crucial role in facilitating the correct protein conformation during the pathogenesis of PD [32].

HSP70 and HSP90 represent a diverse group of highly conserved molecules critical in the cellular stress response to maintain protein homeostasis [33]. HSP90 interacts with α-syn and can influence its conformation and stability. Modulating the activity of HSP90 can influence α-syn aggregation and the formation of Lewy bodies [34].

Increasing HSP70 chaperones can attenuate α-syn aggregation and reduce the toxicity of misfolded proteins [35,36]. Recent research has shown that co-chaperone BAG3 interacts with HSP70 and forms a complex involving sequestosome 1 (SQSTM1), potentially contributing to the activation of macroautophagy and the clearance of α-syn [37]. Conversely, inhibiting HSP70 chaperones can increase the accumulation of protein aggregates [38]. Furthermore, the interaction between HSP70 and other chaperones, such as DNAJ/HSP40 and HSP90, is observed to be essential to maintaining the pool of α-syn in a properly folded conformation [39]. DNAJ/HSP40, a co-chaperone, assists HSP70 by recognizing misfolded proteins and contributing to the degradation of damaged proteins [40].

HSP27 also acts as a chaperone, contributing to the protection of neuronal cells against oxidative and cellular stress [41]. This can reduce the aggregation of α-syn and shield cells from the damage caused by this pathological protein [42].

Protein homeostasis, often referred to as proteostasis, is essential for maintaining the intracellular pool of “healthy” proteins [43]. This process is particularly crucial for neuronal cells, because their proteostatic machinery declines with aging, leading to the accumulation of impaired organelles and misfolded proteins [44,45]. Damaged or non-functioning proteins are tagged with a small protein called ubiquitin, initiating a process known as ubiquitination. This process signals the proteasome to recognize and degrade these proteins [46]. An excess of proteins and an accumulation of misfolded proteins can trigger the onset of the neurodegenerative process in PD [47,48]. Notably, Lewy bodies containing ubiquitinated α-syn have been identified in the brains of individuals with PD. Impaired proteasomal function and genetic findings provide evidence of dysfunction in the ubiquitin–proteasome system (UPS) in PD [49]. Specifically, the accumulation of misfolded α-syn has been suggested to cause UPS dysfunction in dopaminergic neurons in vivo, particularly during the early stages of PD [50]. In this context, stress proteins participate in clearing protein aggregates by initiating autophagy and proteasomal pathways [51,52].

### 3.2. ER Stress in PD Pathogenesis

Other molecular mechanisms implicated in the pathogenesis of PD include ER dysfunction and ER stress [53]. The ER is responsible for the biosynthesis of lipids and steroid hormones, and it serves as a primary site for the accumulation of calcium ions [54]. When the proteins fail to fold correctly, they are translocated into the cytosol through the ER-associated degradation pathway (ERAD) and subsequently degraded by proteasomes [55]. The accumulation of aberrant proteins can induce ER stress, disrupting cellular functions and the secretory pathway [56]. This results in the translocation of glucose-regulated protein-78 (GRP78) within the ER lumen, activating three main transducers: inositol-requiring transmembrane kinase/endoribonuclease 1α (IRE1α), activating transcription factors 6 (ATF6), and protein kinase R-like ER kinase (PERK) [57]. The activation of these factors leads to the expression of target genes associated with apoptotic and adaptive signals that regulate cellular homeostasis, including the chaperone GRP78. This mechanism is referred to as the unfolded protein response (UPR) [58]. It has been demonstrated that an accumulation of misfolded proteins, which cannot be efficiently cleared, results in cytotoxicity mediated by the UPR [59].

This correlation has been established through studies using pharmacologically induced neurotoxic models of PD, where compounds such as 1-methyl-4-phenyl-1,2,3,6-tetrahydropyridine (MPTP), 6-hydroxydopamine (6-OHDA), or rotenone (ROT) have been observed to stimulate UPR genes [60,61]. The involvement of ER stress in PD is further confirmed by experiments in which mice lacking C/EBP homologous protein (CHOP) were protected against 6-OHDA-induced damage, with a reduction in apoptosis and neurons loss [62]. CHOP, also known as growth arrest and DNA damage-inducible protein 153 (GADD153), is a member of the C/EBP transcription factor (CCAAT/enhancer-binding protein) family. The activation of CHOP is widely recognized as a central pathway for apoptosis induced by ER stress [63]. Moreover, evidence of ER stress activation is also evident in the brains of individuals with PD, where the accumulation of ER chaperones within Lewy bodies is observable in the dopaminergic neurons of the substantia nigra [64].

Protective mechanisms against ER stress involve genes such as Parkin and leucine-rich repeat kinase 2 (LRRK2), which are associated with familial PD cases. The overexpression of parkin protects cells from ER stress by activating X-box binding protein 1 (XBP1) through a splicing process, thereby inducing a pro-survival UPR response [65]. According to a recent study, LRRK2 mutations could interfere with the degradation of α-syn by affecting chaperone-mediated autophagy processes in the LRRK2^R1441G^ knock-in mouse model of PD. As age advances, this impairment could lead to a progressive accumulation of toxic α-syn oligomers in the brain [66]. Chaperone-mediated autophagy is essential for facilitating the clearance of selective proteins within neurons. Several lines of evidence have revealed that impairment of the chaperones involved in this process, including HSC70, HSP90, LAMP2A, and PARK7/DJ1, could be related to the pathogenesis of PD [67].

### 3.3. Immune Response in PD Pathogenesis and Possible Contribution of HSP in Peripheral Immune Processes

In PD, neuroinflammation emerges as a hallmark in the early stage of the disease, preceding neuronal loss [68].

During ER stress, some of the target genes stimulated through the UPR play an important role in the inflammatory pathways essential for innate immunity, including nuclear factor kappa-light-chain-enhancer of activated B cells (NF-κB), mitogen-activated protein kinase (MAPK), c-Jun N-terminal kinase (JNK), and p38 [69,70,71]. In response to pathological stimuli, microglia can be activated, releasing pro-inflammatory cytokines, such as tumor necrosis factor alpha (TNF-α) and interleukin-1 beta (IL-1β) [72]. These cytokines can attract additional immune cells, thus amplifying the inflammatory response. Chronic ER stress in neurons predominantly triggers apoptosis, but prolonged ER stress in glial cells can promote a characteristic inflammatory microenvironment in neurodegenerative diseases [73].

Components of the immune system within the nervous central system (CNS), including microglia, astrocytes, and oligodendrocytes, may interact with the immune response of the peripheral nervous system, potentially contributing to the pathogenesis of PD. The role of the peripheral immune system in PD is currently the subject of study and not fully understood. However, the results regarding the interactions between CNS immunity and both innate and adaptive immunity are attracting interest due to their potential for identifying blood biomarkers for the diagnosis of PD [74].

Recent findings suggest that the adaptive immune system plays a significant role in the development and progression of PD. Despite increasing indications suggesting a significant involvement of T cells in the development of the disease, the detailed understanding of how these changes occur at the molecular or cellular levels still requires further research. Arlehamn et al. observed that specific T cells reacting to α-syn were detected many years before the clinical diagnosis of motor PD, suggesting that immunity may have a role in the early stages of the disease [75]. It has even been hypothesized that γδ^+^ T lymphocytes may play a role in targeting HSPs during PD. Fiszer et al. provide evidence of interaction between HSPs and the humoral response in the context of PD. Elevated levels of IgG antibodies against HSPs, particularly HSP70 and HSP65, have been reported in the cerebrospinal fluid and serum of PD patients compared with individuals with other non-inflammatory neurological diseases. The increased antibody levels may be due to a response to stress, previous infections, or cross-reactivity with bacterial or other proteins. These authors suggested a potential connection between HSPs and the immune response in PD that is not linked to the aging process [76]. Bacterial and human HSPs share homology, often leading to cross-reactivity with T cells and antibodies. The presence of anti-self HSP antibodies may be a part of normal immune function, as they are found in healthy individuals. Papuć et al. detected an IgG against HSP60 in the sera of both PD patients and healthy controls. However, the chronic neurodegenerative processes associated with PD may further stimulate the humoral response, increasing anti-HSP60 autoantibody levels in PD patients compared with healthy subjects [77]. The prolonged inflammation has been linked with the autoimmune response and the increase in HSPs. When HSPs interact with cell surface receptors, they may trigger both innate and adaptive immune responses. For example, HSP70 has been found to create an immunogenic complex acting as an enhancer of antigen presentation through major histocompatibility complex class I (MHCI) [77].

Understanding the interplay between peripheral immunity, HSPs, and neuroinflammation, could help in the development of novel diagnostic and therapeutic approaches. The aggregation of α-syn has been found to stimulate microglial activation, contributing to the death of dopaminergic neurons [78]. Immunization with α-syn has been demonstrated to decrease the accumulation of α-syn, while promoting the mobilization of Treg cells and antibodies against α-syn aggregates [79]. In this context, several groups have also reported the potential neuroprotective role of immunotherapy. Labrador-Garrido et al. showed that α-syn associated with specific HSPs could influence the immune response both in vitro and in vivo. This suggested an immunological function for HSPs in addition to their classical chaperone activity [80,81]. Villadiego et al. were the first to demonstrate that α-syn vaccination had the ability to suppress persistent microglial activation in the substantia nigra and striatum during PD. The researchers developed a vaccination based on a complex α-syn with the chaperone GRP94. This approach facilitated the specific targeting of α-syn, ultimately preventing chronic neuroinflammation in a mouse model of PD induced by MPTP treatment. Direct immunization with α-syn/GRP94 resulted in cellular and humoral response in the peripheral system. The authors strongly recommended testing α-syn/GRP94 vaccination following MPTP treatment to assess its potential to revert the existing neuroinflammatory condition [82].

**Figure 2:**
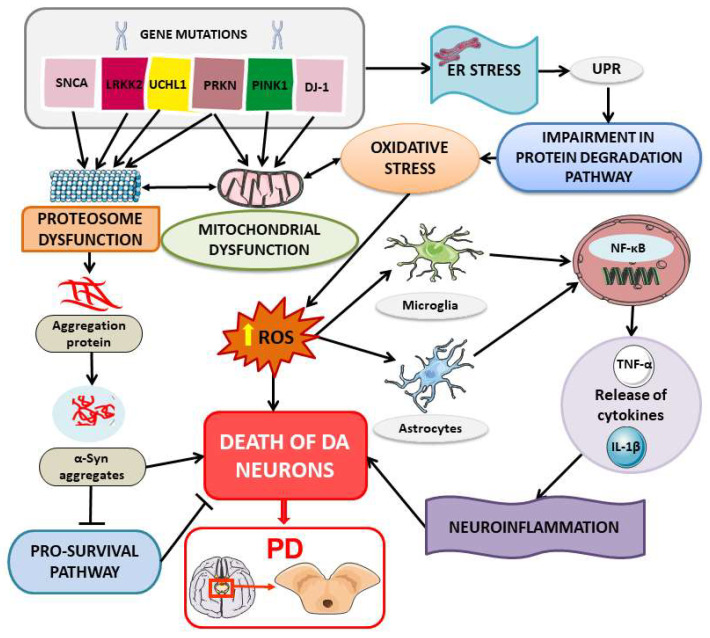
Heat shock proteins and their molecular mechanisms involved in PD pathogenesis. Stress proteins interact with several proteins involved in the processes of PD, such as α-syn and LRRK2. Mutations in PD-related genes can affect the interaction between these proteins and the HSP, altering the HSP’s ability to prevent protein aggregation or facilitate proper folding. Compromised HSPs may not effectively prevent α-syn aggregation, leading to the accumulation of toxic protein aggregates typical of the disease. Defective HSPs may not sustain the efficiency of the UPS, causing the accumulation of damaged proteins and promoting protein aggregation. Furthermore, altered HSPs may fail to mitigate oxidative stress, leaving cells vulnerable to damage caused by free radicals and reactive oxygen species (ROS). This could contribute to persistent inflammation, amplifying the inflammatory process that contributes to neuronal degeneration. Overall, dysfunctional HSPs could compromise cell survival, accelerating the death of neuronal cells in the context of PD pathogenesis. The image was created using the image bank of Servier Medical Art (available online: http://smart.servier.com/, accessed on 15 September 2023), licensed under a Creative Commons Attribution 3.0 Unported License (available online: https://creativecommons.org/licenses/by/3.0/, accessed on 15 September 2023). PD: Parkinson’s disease; SNCA: synuclein alpha; LRRK2: leucine-rich repeat kinase 2; UCHL1: ubiquitin C-terminal hydrolase L1; PRKN: parkin; PINK1: phosphatase and tensin homolog-induced kinase 1; α-Syn: alpha synuclein; ER: endoplasmic reticulum; UPR: unfolded protein response; ROS: reactive oxygen species; NF-κB: nuclear factor kappa-light-chain-enhancer of activated B cells; TNF-α: tumor necrosis factor alpha; IL-1β: interleukin-1β.

In summary, the molecular mechanisms through which stress proteins affect the pathogenesis and progression of PD are complex and interconnected (Figure 2). The accumulation of misfolded proteins, oxidative stress, ER stress, inflammatory response, and mitochondrial dysfunction constitute some factors that contribute to neuronal degeneration and the formation of disease symptoms.

## 4. Therapeutic Approaches for Modulating Stress Proteins in PD

Treatment approaches that modulate stress proteins, such as chaperone-inducing drugs, proteasome inhibitors, antioxidants, gene therapies, stem cell treatments, and gene therapy, are becoming increasingly promising to ameliorate the clinical conditions of Parkinson’s and prevent PD progression. Our body has difficulty recognizing and defending itself against the proteins responsible for neurodegenerative diseases. Therefore, the use of therapies that enhance the stress response aimed at neutralizing the accumulation of proteins associated with this type of disease can be effective [83]. Therapeutic strategies for modulating stress proteins should include more than just pharmacological or genetic methods; they should include the adoption of a healthy lifestyle [84]. Noteworthily, the incidence of PD is influenced by the interaction between genetic and environmental factors that regulate the stress response [85]. Regular exercise, a balanced diet, and correct stress management can mitigate the cellular response to stress and inflammation [84]. Exercise can enhance synaptic strength, potentiate functional neural circuitry, and improve brain plasticity, which is useful for neural rehabilitation in PD patients. It is generally accepted as an intervention that could help address both the motor and non-motor complications of PD and may be considered as a fundamental element of any rehabilitative approach [86].

Due to their role in proteostasis, chaperones can be therapeutically used at any step of protein processing [87]. Using drug compounds aimed at enhancing chaperone expression and/or function has shown promising results in reducing the accumulation of α-syn, as highlighted in several preclinical studies [35,88]. However, whereas increasing HSPs’ activity or levels might have the potential to manage protein aggregation diseases, their efficacy might be compromised in aged tissues [19]. In response to environmental stress and excessive protein accumulation, cellular systems [36] and mouse models [89] have shown a significant increase in chaperone levels, specifically Hsp70.

### 4.1. HSP Inducers

Several therapies employed for PD treatment may directly impact the stress protein expression [90]. Research has shown that the exogenous administration of HSP70 reduces oxidative stress and lipid peroxidation in a cell model of PD. This treatment also increases the levels of various antioxidant biomarkers and protects cells from apoptosis and mitochondrial dysfunction. It has the potential to counter oxidative stress and neuroinflammation in PD [91]. Finally, it was observed that HSP70 also reduced the levels of NF-κB and signal transducer and activator of transcription 3 (STAT3) protein expression [91]. Additionally, experiments with astrocytes exposed to mutated α-synuclein aggregates demonstrated the anti-inflammatory effects of HSP70. In astrocytes, overexpression of HSP70 reduced the expression of several inflammatory proteins, including glial fibrillary acidic protein (GFAP), cyclooxygenase-2 (COX-2), inducible nitric oxide synthase (iNOS), TNF-α and IL-1β. HSP70 exerted anti-inflammatory effects through inhibition of the JNK and NF-κB signaling pathways [92].

Administering recombinant inducible human Hsp70i in an in vivo PD model protected dopaminergic neurons and improved behavioral parameters. It also restored tyrosine hydroxylase levels in neurons, showing its importance in PD treatment [93]. In *Drosophila melanogaster* fruit flies, overexpression of HSP70 protected dopaminergic neuronal cells from neurotoxicity caused by paraquat exposure. This overexpression reduced ROS generation, countered oxidative stress, prevented apoptosis, and improved locomotor deficits and survival [94].

Small molecules called HSP inducers have emerged as possible therapeutic candidates. These molecules stimulate HSP expression by inducing specific cellular pathways [95,96]. The role of HSP70 (HSPA1A) in the dopaminergic nerve cells of the substantia nigra was investigated through a “knock-down” method. Researchers then pharmacologically activated heat shock transcription factor 1 (HSF-1) and increased the expression of HSP70 with a derivative called U-133. These inducers were found to reverse neurodegeneration and increase the number of neurons containing tyrosine hydroxylase, showing promise in slowing down PD-related neurodegeneration [97]. Carbenoxolone, a lipophilic compound, can penetrate the blood–brain barrier [98] and stimulate HSPs [99] such as HSP27.

Co-administration of ROT and carbenoxolone led to the upregulation of HSP70, HSP40, and HSP27 in the midbrain. This resulted in reduced α-synuclein aggregation, enhanced proteasome activity, and increased dopamine levels [100]. These findings suggest promising future directions for potential therapeutic interventions in Parkinson’s disease, particularly in countering oxidative stress, inflammation, and neurodegeneration. The use of HSP inducers and lipophilic compounds may provide new avenues for treatment.

Researchers have explored the role of cysteine string protein α (CSPα), a member of the HSP40 type 3 family, in the context of neuronal survival. CSPα is involved in maintaining the pre-synaptic terminal [101]. The study focused on its phosphorylation by protein kinase C (PKC) and its impact on neuronal health. The study found that when CSPα is phosphorylated at specific serine residues (Ser10 and Ser34) by PKCγ, it promotes the activity of the HSP70 chaperone. This phosphorylation seems to play a protective role in the pre-synaptic terminal and neuronal cells. In contrast, animal knockouts for PKCγ showed a dopaminergic neuronal loss in the substantia nigra and symptoms of PD. In addition, CSPα regulated the survival of PC12 cells, which had been decreased in the CSPα knockdown cells. The CSPα phosphorylation could play an important role in maintaining the normal conformation of synaptosomal-associated protein (SNAP) 25 through the chaperone activity of HSC70/HSP70 [102]. The study’s use of in vitro models, such as PC12 cells, helps elucidate the details of CSPα’s role in neuronal survival. This allows for a closer examination of the cellular processes influencing the survival of dopaminergic neurons. In contrast, the in vivo results provide valuable insights into the relevance of CSPα phosphorylation in a living system. The phosphorylation of CSPα and its impact on HSP70 chaperone activity represent a potential therapeutic target. The results from animal models lacking PKCγ underscore the significance of this protein in maintaining neuronal health. To make this research more applicable to human patients, future studies should focus on translating these findings into clinical settings. Clinical trials should be designed to assess the safety and efficacy of targeting CSPα phosphorylation for the treatment of PD and related conditions.

Recent studies have shown that HSP70 exhibits a specific binding affinity for an amino-terminal segment of a human diffusible survival evasion peptide (DSEP), called dermcidin. This sequence includes CHEC-9, a peptide known for its anti-inflammatory and cell survival properties that has been reported as a potential candidate for treatment of neurodegenerative diseases such as PD [103,104]. In this regard, a preclinical study evaluated the effect of the CHEC-9 peptide binding HSP70 in the cytosol of the cerebral cortex after oral delivery in normal rats. Research suggested that the CHEC-9 peptide increased HSP70 monomers in the frontal cortex of rats, increasing the ability to target misfolded proteins by targeting the ATPase site adenosine triphosphatase (ATPase). Additionally, it was observed that the increased HSP70 monomers that the CHEC-9 peptide induced could affect the larger aggregates for glyceraldehyde 3-phosphate dehydrogenase (GAPDH) [105], an important protein involved in oxidative-stress-induced brain damage and neurodegeneration [106]. This approach has the advantage of targeting the balance between oligomers and monomers of HSP70, which is particularly relevant in diseases such as PD that are characterized by the accumulation of age-related proteins. However, the study primarily used animal models and more research is needed to evaluate its translation to clinical settings [105]. Apelin-13 is another neuropeptide; it acts as a ligand for the orphan G protein-coupled apelin receptor (APJ) [107] and has shown neuroprotective effects in a cellular model of PD using SH-SY5Y cells treated with 1-methyl-4-phenylpyridine (MPP^+^) [108]. This can be cleaved into bioactive peptides, such as apelin-13 and apelin-36 [109]. In particular, pretreatment with apelin-13 reduced ER stress through the inhibition of GRP78/CHOP/cleaved caspase-12 activation and the upregulation of phospho-extracellular signal-regulated kinase 1/2 (ERK 1/2), consequently reducing MPP^+^-induced apoptotic processes [110]. Zhu et al. explored apelin-13′s impact on dopaminergic neurodegeneration and α-syn aggregation in MPTP-treated mice. Apelin-13 alleviated motor impairments in PD mice and protected dopaminergic neurons from MPTP-induced toxicity by reducing α-syn expression. Additionally, apelin-13 reduced the levels of p-IRE1α, XBP1s mRNA and XBP1 protein, CHOP, and GRP78 [111]. In another study, the same research group demonstrated that apelin-36 injection reduced MPTP-induced neurotoxicity in mice and decreased MPP^+^-induced cytotoxicity in cells. Furthermore, α-syn expression decreased in SH-SY5Y cells exposed to MPP^+^ after apelin-36 treatment. These findings were attributed to the downregulation of GRP78, CHOP, and cleaved caspase-12 in both MPP^+^ cells and MPTP mice after apelin-36 treatment, indicating that apelin-36 inhibited ER-stress-mediated cell death [112]. GRP78 (also known as Bip or HSPA5), a HSP70 family ATPase, is an important molecular chaperone and a marker of ER stress that has shown a key role in UPR activation upon the accumulation of misfolded proteins, aiding cell survival [113,114]. Researchers investigated the role of miR-384-5p and GRP78 in regulating ER stress in a cellular model of PD [115]. GRP78 overexpression is known to reduce α-syn-induced neurotoxicity by downregulating ER stress [116]. Indeed, in this study, it was observed that inhibition of miR-384-5p protected cells from toxicity by increasing GRP78 expression. These results highlight the potential of miRNA-based therapies to modulate HSPs and protect neuronal cells from PD-related toxicity [115]. However, the study remains preclinical and further investigations are needed to determine its clinical relevance. Research has also explored the potential of neurotrophic factors as therapeutic agents in the context of neurodegenerative diseases such as PD. Mesencephalic astrocyte-derived neurotrophic factor (MANF) is a promising growth factor for dopaminergic neurons due to its protective effects against ER stress [117]. Research has shown that MANF treatment led to the upregulation of HSP70 and GRP78, resulting in a reduction in 6-OHDA-induced apoptosis in SH-SY5Y cells [118]. In another study, MANF was found to increase GRP78 levels using the same in vitro models of PD. The overexpression of GRP78 was associated with the amelioration of ER stress, significantly inhibiting cleaved caspase-3 [119]. These findings suggest that MANF could exert protective effects against ER stress, making it a potential therapeutic candidate. Cerebral dopamine neurotrophic factor (CDNF), like MANF, is found in the ER and can regulate ER stress [120]. In a study by Voutilainen et al., it was observed that CDNF’s impact on ER stress demonstrated a potential neurorestorative effect, both independently and when combined with glial cell line-derived neurotrophic factor (GDNF) protein in a rat model of PD. This evaluation was performed in both dopaminergic neuron cultures and in the midbrains of rats after 6-OHDA injury. CDNF treatment activated the phosphoinositide 3 kinase (PI3K)/serine-threonine kinase (AKT) pathway; it reduced GRP78 and the phosphorylation of eukaryotic initiation factor 2α subunit (eIF2α) levels both in vitro and in vivo. In contrast, GDNF alone activated the ERK1/2 and AKT pathways but had no effect on ER-stress-related proteins. Additionally, combining CDNF with GDNF appears to be more effective in restoring the dopaminergic function in rat models of PD [121]. These studies open exciting possibilities for combination therapies in the treatment of this condition. However, whereas these results are promising, clinical trials and further in vivo studies are necessary to evaluate the translational potential and safety of these neurotrophic factors as therapeutic options for neurodegeneration. Future investigations should aim to optimize the delivery methods, assess the long-term safety, and explore potential synergistic effects when combining different neurotrophic factors.

### 4.2. HSP90 Inhibitors

On the other hand, Hsp90 inhibitors have been investigated in several PD models because their repression has shown a promising protective effect against neurodegeneration [122]. Inhibiting HSP90 leads to an increase in the expression of protective HSPs, such as Hsp70, as it is part of a complex that negatively regulates the activity of HSF-1s [123]. Consequently, there is growing interest among researchers to identify small molecule HSP90 inhibitors with improved pharmacokinetics, including BBB permeability [88]. Several of these novel Hsp90 inhibitors, in particular SNX-0723(4-[6,6-dimethyl-4-oxo-3-(trifluoromethyl)-4,5,6,7-tetrahydro-1H-indazol-1-yl]-2-[(trans-4-hydroxy cyclohexyl)amino]benzamide; PF-04928473), significantly reduced α-syn oligomer formation and cytotoxicity concomitant with HSP90 inhibition and HSP70 induction [124]. Based on these findings, an in vivo study using a PD rat model showed that oral administration of SNX-0723 (PF-04924868); and SNX-9114 (PF-04944733) prevented progressive α-syn-induced nigrostriatal toxicity. These data confirm that targeting HSP90 and increasing the cellular response to stressors could be a promising therapeutic approach to protect against neurodegeneration [125].

In this context, Behrang Alani et al. tried to understand how HSP90 inhibition could influence cellular processes in an in vitro model of PD. In 6-OHDA-exposed PC2 cells, the use of a siRNA to suppress the expression of Hsp90 inhibited pro-apoptotic factors such as Bax, caspase-3, and PARP, while increasing the expression of Bcl2 (an anti-apoptotic factor). This confirmed that silencing HSP90 inhibited 6-OHDA-induced apoptosis. The silencing of HSP90 caused the dissociation of Keap1 and HSP90 from nuclear factor erythroid 2-related factor 2 (NRF2) and HSF-1, respectively. NRF2 enters the nucleus and upregulates antioxidant enzymes, thus protecting cells from the oxidative stress induced by 6-OHDA, like in PD. The nuclear translocation of HSF-1 leads to enhanced expression of genes such as HSP90 and HSP70 [126].

These results emphasize the significance of HSP90 inhibition and its connection to histone deacetylase 6 (HDAC6), which plays a role in activating heat shock factor 1 (HSF1) and subsequently inducing the expression of crucial cellular chaperones [13,127,128]. HDAC6 regulates cellular response pathways towards ubiquitinated cytotoxic aggregates in some neurodegenerative diseases as it deacetylates non-histone substrates such as α-tubulin and HSP90 [129]. Yunlan Du et al. investigated how HDAC6 affects the α-syn aggregation in PD models by triggering the heat shock response. In the UPS-impaired mouse model of PD, inhibiting HDAC6 showed potent inhibitory effects on HDAC activity. However, it worsened dopaminergic neurodegeneration and increased the levels of α-syn oligomers in the nigrostriatal region. In contrast, in vitro experiments indicated that overexpression of HDAC6 protected cells from α-syn-induced toxicity, suggesting that HDAC6-mediated dissociation between HSP90 and the transcription factor HSF1 led to HSF1 activation [130]. In another study, Janina Leyk et al. showed that HDAC6 was involved in the formation of protein aggregates, as well as autophagy, in oligodendroglial cells. The researchers aimed to understand how inhibiting this protein could impact these processes, particularly when the UPS was inhibited by carbobenzoxy-L-leucyl-L-leucyl-L-leucinal (MG-132). To do this, they treated primary cultures of rat brain oligodendrocytes with tubastatin A and HDAC6 small hairpin RNA (shRNA). In the in vitro experiments, the inhibition of HDAC6 did not prevent the accumulation of protein aggregates induced by MG-132. This failure to counteract aggregate formation led to cell death. Additionally, the inhibition of HDAC6 resulted in the suppression of HSP70 activity, thereby altering the cellular stress response. Further investigations using an oligodendroglial cell line (OLN-93) that was stably transfected with the longest human tau isoform (OLN-t40) and labeled with a green fluorescent protein (GFP) linked to the microtubule-associated protein 3 (LC3) light chain were conducted. Inhibition of HDAC6 in this setting led to the accumulation of LC3-positive autophagosomal vacuoles [131]. However, when the role of HDAC6 was evaluated in a rat model of PD, the results were different. In this case, the inhibition of HDAC6 using tubastatin A resulted in increased levels of chaperone proteins such as Hsc70 and Lamp2 while reducing the levels of α-syn [132]. The noteworthy difference between the in vitro and in vivo results underscores that whereas targeting HDAC6 in PD models shows promise in some contexts, it appears to worsen neurodegeneration in other situations. This discrepancy underscores the complexity of neurodegenerative diseases, the difficulty of translating findings from cell-based studies to living organisms, and the need for more precise and context-specific therapeutic strategies. Future research should focus on elucidating the factors that determine the differential effects of HDAC6 inhibition in various cellular and animal models. Furthermore, understanding the precise mechanisms by which HDAC6 modulates the stress response and protein aggregation could pave the way for the development of more effective and targeted therapeutic approaches for PD.

### 4.3. HSP-Modulation-Based Gene Therapy for PD Management

Gene therapy could be a viable non-pharmacological therapeutic approach to reduce stress by genetically altering targeted cells to directly target the accumulation of misfolded proteins or induce the overexpression of chaperones [133].

An experimental study highlighted the important role of pharmacological or genetic activation of the chaperone function in counteracting the proteasomal and mitochondrial dysfunction in neurodegenerative conditions. Notably, HSP70 overexpression in SH-SY5Y cells, achieved either by stably expressing human HSP70 or inducing it through 17-AAG treatment, demonstrated protective effects against proteasome dysfunction. This effect was similar to the overexpression of parkin, a key regulator of mitochondrial homeostasis and essential for dopaminergic neuronal survival [134]. It is worth noting that parkin mutations are associated with familial PD and contribute to proteasome dysfunction [135]. Chaperones exhibited compensatory properties, even mimicking parkin’s function when tested in Drosophila parkin null mutants *24B-Gal4* (muscle-specific) and *UAS-Hsp70* (*UASHsap/HSPA1L.W Bonin*). The advantages of these findings include the potential to harness gene therapy to manipulate chaperone expression and combat protein misfolding and proteasome dysfunction in neurodegenerative diseases. Additionally, the chaperones’ ability to mimic parkin’s function suggests their adaptability and effectiveness in different models. However, some challenges remain, such as the need for more extensive studies to confirm these results in several models of PD and to understand the precise mechanisms underlying chaperones’ compensatory actions [134].

In this regard, it was examined how overexpression of the carboxyl terminus of Hsc70-interacting protein (CHIP) in *Drosophila* flies could influence the symptoms and mitochondrial defects associated with mutations in the *PINK1* and *PARK* genes that are known to be implicated in PD [136]. CHIP is a chaperone-associated E3 ligase that targets HSP70 and HSP90 for degradation [137]. It is also known that it forms a complex with HSP70 and parkin to maintain protein homeostasis and facilitate protein folding or degradation [138]. In *Drosophila* flies, genetic mutations in the *PINK1* and *PARK* genes resulted in various symptoms, including wing issues, locomotion problems, muscle degeneration, and dopaminergic neuron loss, accompanied by mitochondrial abnormalities and a reduced ATP content. However, when CHIP was overexpressed in these mutant flies, it mitigated these symptoms and improved mitochondrial function [136]. In separate in vitro experiments, the researchers manipulated CHIP expression using various methods, including CHIP-WT plasmid transfection and recombinant adeno-associated virus (AAV) delivery. Overexpressing CHIP protected cells from MPP^+^-induced damage and alleviated motor deficits and dopaminergic neuron loss in MPTP-treated mice. Furthermore, the modulation of CHIP reduced α-syn aggregate toxicity in rats [139]. The effect of the modulation of CHIP on α-syn aggregate toxicity was also evaluated by Dimant et al. in SD rats co-injected unilaterally with a new viral vector able to directly detect α-syn aggregates. Post-mortem microscopic analyses revealed that co-expression of CHIP led to a reduction in α-syn aggregates in rat brains that involved tyrosine hydroxylase degradation [140]. The advantages of these studies lie in the potential of CHIP overexpression as a therapeutic approach for PD by targeting mitochondrial and protein homeostasis-related issues. This approach appears promising in both in vivo and in vitro models of PD. However, some challenges include the need for further investigations to understand the long-term effects and safety of manipulating CHIP expression.

Thus, for gene delivery, the use of viral vectors such as AAV and lentivirus is certainly a more efficient approach than the use of non-viral vectors [141]. Through the stereotactic injection of recombinant viral vectors, short RNA molecules, such as small interfering RNA (siRNA) and shRNA, can be targeted precisely to specific brain regions [142]. Although gene therapy is still under investigation, encouraging outcomes from concluded and ongoing clinical trials are guiding the refinement of this approach [143]. This may offer the advantages of early detection and treatment to slow or halt the progression of nigrostriatal neurodegeneration [144].

In this regard, Teresa C. Moloney et al. evaluated the effects of AAV-mediated overexpression of HSP27 and HSP70 in preventing α-syn-induced toxicity. Sprague Dawley mice were co-injected intranigrally with pathogenic viral vectors (AAV-α-syn) and with AAV-HSP27 or AAV-HSP70. In this study, AAV-mediated overexpression of HSP70 demonstrated significant neuroprotective effects in the α-synuclein-induced PD model. It preserved the integrity of nigrostriatal dopaminergic neurons and reduced α-synuclein accumulation. In contrast, HSP27 overexpression did not protect animals from α-synuclein-induced pathology, suggesting differences in the protective potential of several heat shock proteins [145]. Administering purified recombinant HSP70 through intranasal delivery in the 6-OHDA rat model showed neuroprotective effects, reducing neurotoxicity and neuroinflammation. Microglial activation and astrogliosis were decreased, indicating a potential role for HSP70 in alleviating neuroinflammatory responses [146].

The research investigated the role of the ER chaperone protein GRP78 in aging and PD pathogenesis. Lower levels of GRP78 were observed in aged mice compared with young mice. Reduced GRP78 expression worsened α-synuclein-induced neurotoxicity. These data suggested that GRP78 plays a role in aging and may contribute to PD progression, highlighting its potential as a therapeutic target [147]. These studies highlight the potential of gene therapy, particularly through AAV-mediated approaches, for the management of PD. Additionally, they emphasize the significance of specific chaperone proteins such as HSP70 and GRP78 in mitigating neurodegenerative processes and neuroinflammation. Future studies should explore the translation of these preclinical findings into clinical settings.

Sertan Arkan et al. studied the effects of DNAJB6 overexpression in HEK293 cells and in rats using wild-type adeno-associated viral (AAV6) vectors designed to overexpress human α-syn and DNAJB6 [148]. DNAJB6 is a member of the HSP40/DNAJ family, acts as a co-chaperone in the HSP70 cycle and, at the same time, plays an independent role in preventing protein aggregation. In human diseases, mutations in eight distinct J proteins (DNAJB2, DNAJB6, DNAJC5, DNAJC6, DNAJC12, DNAJC13, DNAJC19, and DNAJC29) have been described [149]. *DNAJB6* is one of the most recent genes identified as being responsible for autosomal recessive hereditary/juvenile [150]. This chaperone plays a major role in protein folding and quality control as well as in the pathogenesis of PD [151]. In cells, DNAJB6 prevented α-synuclein aggregation. In rats, it counteracted cell death and improved behavioral deficits induced by α-synuclein overexpression. DNAJB6 may interfere with protein aggregation in the early stages of PD pathogenesis, suggesting its potential as a therapeutic target for PD management [148]. These data encourage the use of viral vectors in humans to modulate HSPs such as DNAJB6, which represent a potential therapeutic target for PD management. In this regard, researchers examined the role of CSPα, another DNAJ/HSP40 family member, in synaptic aggregation in PD. Overexpressing CSPα in PC12 cells and in 1-120hαSyn mice restored synaptic vesicle recycling and normal dopamine release, alleviating the synaptic aggregations of α-synuclein. Modulating CSPα represents a potential therapeutic strategy for restoring normal synaptic function, particularly in the early stages of PD [152]. Another chaperone molecule potentially used in gene therapy is HSP104, a conserved AAA+ hexameric yeast protein known to solubilize protein aggregates [153]. Jackrel et al. engineered a highly active Hsp104 mutant that disassembles pre-formed protein aggregates from pre-existing inclusions. In this study, the researchers modified Hsp104 through specific mutations in certain regions of the protein, such as helix 1, 2 (Hsp104^A437W^) or in helix 3 (Hsp104^A503V^ or Hsp104^Y507C^). In yeast, these mutants, compared with wild-type HSP104, more effectively reduced protein aggregates, promoted correct protein localization, and reduced the toxicity of aggregated proteins. The authors tested the ability of these HSP104 mutants in a transgenic *Caenorhabditis elegans* (*C. elegans*) PD model. To target the expression of HSP104 and α-syn variants in dopaminergic neurons, the researchers used the promoter of the dopamine transporter gene (dat-1). The mutants significantly protected dopaminergic neurons in worms against α-syn toxicity, maintaining a greater number of dopaminergic neurons [154].

Targeting specific chaperone proteins such as DNAJB6, CSPα, and engineered HSP104 mutants shows promise in mitigating α-synuclein aggregation and related neurotoxicity. These approaches have demonstrated efficacy in both in vitro and in vivo models. Future research in this direction could pave the way for novel treatment strategies. Thus, exploring gene therapy strategies for modulating chaperone proteins is an exciting avenue for potential PD therapeutics.

### 4.4. The Therapeutic Potential of HSPs in Stem-Cell-Based PD Treatments

Human mesenchymal stem cell (hMSC) transplants are aimed at replacing or regenerating damaged brain cells in the brains of PD patients [155]. However, the transplant can cause a kind of “stress” for the transplanted cells, as they must adapt to the surrounding brain environment, settle, and mature into functional neurons. HSPs play a crucial role in protecting transplanted stem cells from environmental stress and ensuring their optimal function. HSPs help prevent protein damage during the transplantation process, which is essential for the success of cell replacement therapy. [156]. The use of human bone-marrow-derived mesenchymal stem cells (hBM-MSCs) has emerged as an alternative strategy to replace or help damaged cells in PD [157]. The transplantation of stem cells is typically performed into the striatum of rats previously lesioned with 6-OHDA [158]. Most of the therapeutic properties of hBM-MSCs may be due to their secretome; the cells are capable of secreting important trophic factors such as brain-derived neurotrophic factor (BDNF), epidermal growth factor (EGF), vascular endothelial growth factor (VEGF), neurotrophin-3, and fibroblast growth factor 2 (FGF-2) [159]. In this regard, Fábio G. Teixeira et al., using previously generated proteomic databases and mass spectrometry-based analyses, have identified in the hBM-MSCs secretome the presence of several neurotrophic factors and cytokines with important roles and therapeutic actions on PD, including HSP27 (HSPB1) [160]. It is known that HSP27 attenuates α-syn-induced toxicity by directly binding to α-syn fibrils, thus preventing its aggregation and protecting dopaminergic neurons against death [161]. Among the molecules potentially involved in the secretome, HSP27 was suggested to protect dopaminergic neurons. Study results showed that intracranially injecting the hBM-MSC secretome directly into the substantia nigra and striatum improved motor deficit and restored the neuronal structure in 6-OHDA rats [160].

To confirm that the use of secretome derivatives could improve the potential problems associated with cell transplantation, in one study, the hBM-MSC secretome was compared with hBM-MSC transplantation in a 6-OHDA mouse PD model. The secretome treatment led to a more significant recovery for dopaminergic neurons and improved animal behavior compared with direct cell transplantation. In vitro, the secretome enhanced neuronal differentiation, suggesting the important role of the secretome in neuronal cell survival compared with hBM-MSC transplantation. In conclusion, the proteomic characterization of the secretome highlighted some molecules linked to the UPS, a component involved in PD pathophysiology. HSPs are believed to influence proteostasis processes, contributing to the neuroprotective effects. Modulating the expression of HSPs in stem cells before transplantation holds promise as an innovative therapeutic strategy for PD and other neurodegenerative diseases [162].

Recently, together with mesenchymal stem cells, neurotrophic factors have shown promise for PD treatment. Neurotrophic proteins exhibit significant neurorestorative and neuroprotective properties in PD [163]. Due to their large size, they cannot cross the blood–brain barrier. Current solutions could include viral vector gene therapy or pre-engineering stem cells with the neurotrophin gene for transplantation [164]. In this regard, in a PD rat model, Jiang et al. evaluated the effects of BDNF-modified umbilical cord mesenchymal stem cells (hUC-MSCs) differentiated into dopaminergic neurons. These cells differentiated into dopaminergic-like neurons and were transfected with BDNF-related vectors. The study reported improved motor behavior, increased neuronal markers, higher dopamine levels, and the upregulation of HSP60, toll-like receptor 4 (TLR4), and myeloid differentiation factor 88 (MyD88) in the substantia nigra and striatum. Significant HSP60 upregulation was noted in the substantia nigra and striatum, suggesting its role in an anti-neuroinflammatory response through microglial activation [165]. In summary, these preclinical studies highlight the promise of secretome derivatives and neurotrophic factors as potential therapeutic strategies for PD. Specifically, using secretome derivatives demonstrates superior effects on neuronal recovery and functional improvements compared with cell transplantation alone. Identifying the role of HSPs in the secretome and their impact on proteostasis processes provides insights into the mechanisms behind these improvements. Neurotrophic factors, such as BDNF, also represent an exciting avenue for neuroprotection and restoration in PD. Overcoming the blood–brain barrier and optimizing the number of transplanted neurons are among the challenges that require further investigation. Further research is needed to refine the use of secretome derivatives and neurotrophic factors in clinical applications. Exploring the potential of modulating HSP expression in stem cells for transplantation holds promise and warrants additional investigations for translational and clinical studies.

**Figure 3:**
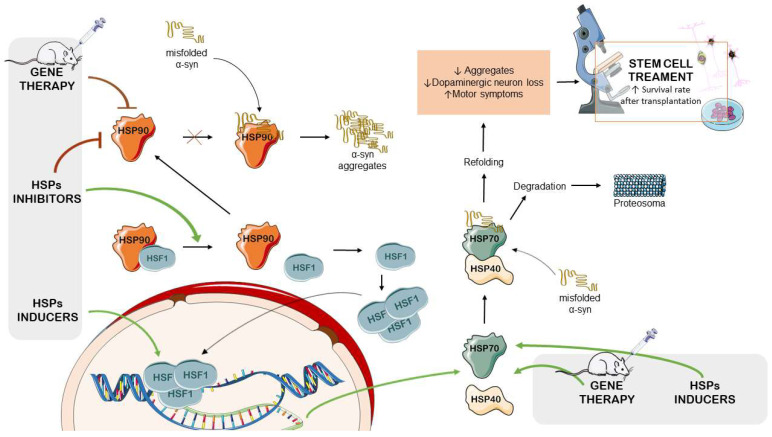
The main therapeutic approaches targeting the modulation of stress proteins to reduce the impact of PD. The simultaneous employment of pharmacological and genetic strategies and stem cell therapies may offer the possibility of protecting neuronal cells, thus preventing the principal etiopathogenesis processes of PD. The image was created using the image bank of Servier Medical Art (available online: http://smart.servier.com/, accessed on 15 September 2023), licensed under a Creative Commons Attribution 3.0 Unported License (available online: https://creativecommons.org/licenses/by/3.0/, accessed on 15 September 2023). HSPs: heat shock proteins; HSF-1: heat shock factor-1; α-syn: alpha-synuclein.

Therapeutic approaches targeting the modulation of stress proteins may have the potential to develop new strategies that could help to reduce the impact of PD (as summarized in Table 1). The simultaneous utilization of pharmacological, genetic, and stem cell treatments may offer the possibility to protect neuronal cells, preventing protein damage and inflammation, which are processes central to the pathogenesis of PD (Figure 3). These studies open avenues for exploring innovative strategies to target stress proteins and combat PD, thus offering hope for improved treatments in the future. These efforts will be essential in bridging the gap between preclinical research and clinical applications, thus offering potential benefits to PD patients.

## 5. Therapeutic Compounds as Potential Treatment in PD: Insights from Preclinical Studies on Stress Protein Modulation

**Figure 4:**
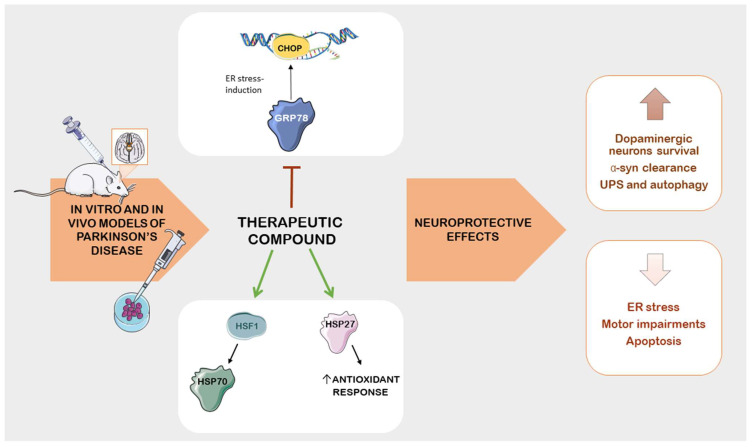
The main HSPs modulated by therapeutic compounds in preclinical models of PD. Therapies, both chemical and natural, regulate pathological changes in PD by targeting specific chaperone proteins and reducing ER stress. Key chaperones involved include GPR78/CHOP, HSF1/HSP70, and HSP27. Modulation of HSPs by therapeutic compounds preserves dopaminergic neurons, enhances α-syn degradation, activates the UPS, and promotes autophagy. Simultaneously, this neuroprotective effect leads to the suppression of ER stress, alleviation of motor deficits, and a decrease in cell death. The image was created using the image bank of Servier Medical Art (available online: http://smart.servier.com/, accessed on 15 September 2023), licensed under a Creative Commons Attribution 3.0 Unported License (available online: https://creativecommons.org/licenses/by/3.0/, accessed on 15 September 2023). HSPs: heat shock proteins; PD: Parkinson’s disease; ER: endoplasmic reticulum; GPR78: G protein-coupled receptor 78; CHOP: C/EBP homologous protein; HSF-1: heat shock factor-1; α-syn: alpha synuclein; UPS: ubiquitin–proteasome system.

Several pharmacological and natural compounds have demonstrated potential in protecting neurons by modulating HSPs in PD models (Figure 4). However, mitigating or slowing down neuron loss in PD presents challenges due to late diagnosis, as early-stage manifestations resemble those of other neurodegenerative diseases [166]. PD is a complex pathology with multiple causal factors; therefore, creating an experimental model that accurately replicates this condition is challenging [167]. To counteract the progressive neurodegeneration in PD, one strategy involves the development of neuroprotective treatments aimed at preserving dopaminergic neurons and restoring dopamine levels [168]. This approach targets stress proteins to ameliorate Parkinson’s pathology and symptoms (as summarized in Table 2). Although the translation of data into practical application has not been successful yet [169], in this section, we will discuss specific therapeutic or natural molecules that preserve nervous system function by modulating HSPs in PD models.

### 5.1. Pharmacological Modulation of HSPs and Neuroprotective Effects in PD Models

Selegiline is a type B monoamine oxidase inhibitor that may be used as a safe and well-accepted drug in PD treatment, either alone or in combination with levodopa. Preclinical data, however, suggest that the protective qualities may not be exclusively linked to its enzymatic inhibitor activity, thus indicating the potential of selegiline as a neuroprotective and neurotrophic agent in PD [170]. Recently, research has revealed that pretreatment with selegiline in rat neural stem cells led to an increase in HSPA4RNA expression, resulting in the mitigation of ROS levels and mitochondrial DNA damage. This upregulation of HSPA4 appears to have antioxidant and anti-apoptotic functions. However, the specific function of HSPA4 in PD is not yet clear [171]. HSPA4 belongs to the HSP70 family, but its role in PD is still unclear [172]. It is believed that it may contribute to the maintenance of mitochondrial homeostasis by interacting with DJ-1, parkin, and PINK1 [173]. One advantage of this approach is that it suggests a potential benefit for selegiline in the context of PD beyond its role as an enzymatic inhibitor. However, there are significant challenges. The in vitro results should be confirmed in vivo using animal models of PD to better understand the role of HSPA4. Additionally, it should be noted that the experimental model of PD may not accurately reflect the real-life condition, which could lead to the limited applicability of preclinical results [174]. Furthermore, because of the absence of clear tools or criteria for assessing disease progression in the brain, clinical trials have not successfully established these effects in patients, thus posing challenges for accurate and reliable clinical trials. Furthermore, the neuroprotective effects of selegiline have not yet been demonstrated in clinical studies. The absence of clear tools or criteria for assessing disease progression in the brain and the heterogeneity of patients mean that clinical trials have not been successfully established, thus posing challenges for accurate and reliable clinical trials [175]. Wang et al. conducted a systematic review and meta-analysis regarding the efficacy and safety of selegiline in the therapy of PD for different treatment durations. The researchers analyzed 27 randomized controlled trials and 11 observational studied to assess the effect of selegiline on the severity and progression of PD in patients. In this context, selegiline appears to have benefits in improving PD symptoms but has a high risk of adverse events, especially neuropsychiatric issues. Further studies are needed to better understand this correlation and assess the long-term efficacy and safety of selegiline [176].

Several preclinical studies have explored the regulation of HSPs to combat PD by enhancing antioxidant defenses and reducing oxidative stress. Some noteworthy findings include lapatinib ditosylate, a tyrosine kinase inhibitor primarily used as an anti-cancer drug, which improved motor deficits and reduced dopamine loss and oxidative stress in ROT-induced rats. Mansour et al. found that lapatinib blocked the activity of the HSP90/CDC37 chaperone complex, leading to improved PD-related histopathological alterations in the substantia nigra of rats. Furthermore, lapatinib had an impact on proteins such as LRKK2, α-syn, and c-ABL, resulting in the restoration of dopamine levels as well as the suppression of ferroptosis and lipid peroxidation. This study highlights the potential of targeting the HSP90/CDC37 complex to activate the antioxidant system [177], although it is crucial to consider its possible impact on glutathione (GSH) levels, which also play a role in PD pathogenesis [178].

Sodium salicylate demonstrated a modulatory effect in preventing protein aggregation and neuronal death in a PD rat model. This was achieved by enhancing GSH levels and proteasome activity. Sodium salicylate induced the activation of heat shock factor 1 (HSF-1) and subsequent upregulation of downstream HSPs, including HSP-27. In particular, HSP27 was associated with preventing caspase activation and dopaminergic neuronal apoptosis. This approach also improved proteasome activity and GSH levels and reduced astrocyte activation, ultimately reducing the aggregation of α-syn and ubiquitin. Sodium salicylate holds promise for neuroprotection through its various pathways, including mitigating neuroinflammation [179]. One advantage of these approaches is their potential to target multiple factors in PD through the regulation of HSPs, enhancing antioxidant defenses and reducing oxidative stress. This presents an intriguing direction in the development of neuroprotective therapies. However, further research is needed to better understand their specific mechanisms, potential side effects, and long-term efficacy, especially in the context of clinical applications.

Rifampicin, an antibiotic traditionally used to treat tuberculosis and leprosy, has shown promise in preclinical studies for its potential neuroprotective effects. These effects are mediated through the regulation of GRP78 in cellular models of Parkinson’s disease (PD), specifically in ROT-treated PC12 cells. Jing et al. demonstrated for the first time that pretreatment with rifampicin provided cytoprotective effects by modulating PERK, eIF2α and ATF4, leading to the activation of GRP78 and UPR. This modulation ultimately led to the activation of GRP78, an endoplasmic reticulum chaperone protein associated with neuroprotection. The authors suggested rifampicin as a potential inducer of GRP78 that could hold therapeutic value in PD. However, it is important to note that in vivo experiments are necessary to validate these findings and further research is required to establish the neuroprotective mechanisms of rifampicin [180]. The advantages of this approach include repurposing an existing antibiotic for a new therapeutic application and potentially providing a cost-effective option for PD treatment. Additionally, targeting UPR pathways and GRP78 activation may offer a novel way to protect neurons from PD-related insults.

On the other hand, other studies have shown that activation of GRP78 pathway might be induced by ROT. The overexpression of ATF4 during prolonged ER stress leads to the activation of CHOP, resulting in neuronal death [181]. This suggests that the role of GRP78 and UPR in PD is complex, context dependent, and requires careful investigation.

Research exploring the antidiabetic drugs empagliflozin and metformin has shown promise in inhibiting ER-stress-related pathways in PD models. Empagliflozin reduced the GRP78 levels, as well as other ER-stress-related proteins (p-PERK, total PERK, eIF2α, and CHOP), in the striatum of ROT-treated rats. In this way, the drug improved α-syn clearance by enhancing autophagy and the UPS, thereby reducing oxidative stress and neuroinflammation. These effects were associated with improved motor deficits in treated rats. The advantage of targeting ER-stress-related pathways with drugs such as empagliflozin is the potential to mitigate PD-related alterations. However, further research is needed to confirm these neuroprotective mechanisms in different PD animal models, including different genders and species [182].

Metformin, an antidiabetic drug, has demonstrated potential neuroprotective effects in preclinical studies related to PD. In mouse models of PD induced by ROT, researchers observed that metformin pretreatment alleviated endoplasmic reticulum (ER) stress, protecting against dopaminergic neurodegeneration. This protective effect was associated with the downregulation of the ER-related genes ATF4, ATF6, XBP1, GRP78, and CHOP. As a result, metformin increased cell viability and improved behavioral impairments. Furthermore, the drug suppressed neuroinflammation by reducing the expression of pro-inflammatory cytokines such as TNF-α and IL-1β, which are linked to microglia activation [183]. Further research is necessary to evaluate its ability to maintain ER homeostasis in models other than the ROT model. Both the antidiabetic drugs seem to inhibit neuroinflammation in ROT-treated animals. However, the neuroprotective effect of metformin on dopaminergic neurons has been previously confirmed in other PD animal models [184]. The advantages of this approach include the repurposing of a widely used antidiabetic medication for potential PD treatment, which offers a well-established safety profile and ease of clinical translation. By modulating ER stress and reducing neuroinflammation, metformin addresses key pathological factors in PD.

In a distinct study, Fitzgerald et al. highlighted the protective effect of metformin in mitigating the pathological R47X mutation in TRAP1, thereby restoring the mitochondrial membrane potential in a human cell PD model. The R47X TRAP1 mutation was found in fibroblasts from a PD patient and was associated with increased levels of the chaperone proteins HSP60, HSP90, and HSP70. The authors linked the loss of TRAP1 function with an increased the UPR within mitochondria and an enhanced mitochondria turnover. The advantage here is the potential to target mitochondrial dysfunction, a crucial aspect of PD pathogenesis [185]. Furthermore, epidemiological data have suggested an association between diabetes and an increased risk of developing PD, making antidiabetic agents such as metformin a promising avenue for counteracting neurodegeneration in PD. Clinical trial results support the idea that metformin can improve cognitive defects in the Parkinson’s population. However, further research is needed to confirm the clinical efficacy of interventions such as empagliflozin in PD patients [186]. Translating these promising preclinical findings into effective clinical treatments requires careful investigation and clinical validation.

### 5.2. Natural Remedies Modulating HSPs for Neuroprotection in PD Models

Several natural compounds that will be reported in this paragraph have demonstrated potential neuroprotective effects and benefits for the management of PD by involving or modulating HSPs. A number of findings highlight the promising role of certain substances and therapeutics agents in addressing the pathological features of PD, such as the accumulation of α-syn proteins, oxidative stress, inflammation, and the loss of dopaminergic cells. The activation of protective HSPs, especially HSP70 and HSP27, could promote the clearance of damaged or aggregated proteins and contribute to the survival of neurons. Furthermore, many of the mentioned substances appear to act to reduce ER stress, thus helping to preserve cell health. These compounds have shown promise as alternative therapies to potentially alleviate PD pathology and symptoms. Many natural compounds are well tolerated and lead to fewer adverse events than synthetic drugs. Their neuroprotective effects, achieved through the modulation of HSPs, may enhance cellular stress tolerance and prevent dopaminergic cell loss (as shown in Figure 3).

#### 5.2.1. The Activation of HSF1/HSP70 by Natural Compounds

Melatonin, a pineal hormone that regulates circadian rhythm, has demonstrated its effectiveness as a therapeutic agent for PD patients, with supporting evidence from experimental models [187]. In vitro studies have suggested that melatonin can increase the levels of HSF1 and HSP70, promoting cell viability and neuroprotection. By pretreating cells with melatonin, researchers induced the HSF1/HSP70 pathway, promoting neuroprotection against the toxicity induced by MPP^+^. Silencing HSF1 through siRNA transfection suppressed the protective benefits on cell survival and antioxidant response induced by melatonin. Consequently, the induction of HSF1/HSP70 may play a role in preventing neurodegeneration in PD by reducing oxidative stress and apoptosis in neurons [188]. The advantages of melatonin as a potential treatment for PD include its ability to induce HSF1/HSP70 activation, which addresses key pathological factors in the disease. However, it is important to note that, in some cases, high doses of melatonin may be required and its precise role in PD treatment remains unclear. From a safety perspective, caution is advised in children due to the potential effects of melatonin on reproductive development, despite limited evidence in humans [189].

The neuroprotective potential of inducing HSF1/HSP70 activation has also been confirmed in other in vitro studies with different substances. For example, glutamine was found to increase HSP70 levels in cells overexpressing α-synuclein, a protein associated with PD. However, HSP70 upregulation was dependent on HSF-1 stimulation, as shown when HSF-1 was silenced. This enhancement, in turn, promoted α-syn degradation [190]. These findings were also confirmed in PC12 cells, where glutamine significantly increased HSP70 mRNA expression and its related protein levels in an HSF1-dependent manner [191]. These findings indicate that glutamine may prevent α-syn aggregation in vitro. Further research is needed to confirm these molecular mechanisms in different preclinical studies. Overall, these studies provide valuable insights into the potential therapeutic strategies for PD by targeting HSF1 and HSP70. However, further research, especially in preclinical models, is needed to confirm these mechanisms and evaluate their translational potential for clinical studies. Additionally, the safety and optimal dosage of melatonin in humans, particularly in specific populations such as children, require further investigation.

Several natural compounds have shown promise in preclinical studies for their potential to promote neuron survival through the upregulation of HSP70, offering a novel therapeutic approach for Parkinson’s disease (PD). Compounds such as sesamol and naringenin, which are flavonoids found in various foods and medicinal plants, have demonstrated neuroprotective, cognitive-enhancing, and motor-control-maintaining effects. Angeline et al. demonstrated that oral administration of sesamol and naringenin increased the expression of HSP70 and HSP90 and inhibited pro-apoptotic caspase-3 and caspase-9 activation, thus preserving nigrostriatal integrity and neuronal survival and improving muscle health. Notably, HSP70 and HSP90 remained unaltered in muscle tissues, whereas the downregulation of the mitochondrial chaperone HSP60 in ROT-treated animals was restored after sesamol treatment. Additionally, a decrease in the ubiquitin level suggested the clearance of harmful proteins, highlighting the ability of flavonoids to provide neuroprotection and support neurorecovery [192].

Spirulina, a nutrient-rich blue-green algae biomass, modulated HSPs in a PD fly model. *DJ-1β^Δ93^* flies, a transgenic PD model in *Drosophila*, showed increased symptoms when exposed to oxidative stress. Spirulina and its active component C-phycocyanin decreased HSP70 and JNK levels, improving survival and enhancing antioxidant defenses. This, in turn, improved the lifespan and mobility of the mutant flies when compared with the wild-type strain *Oregon R*^+^. The observations were performed through immunostaining assay in the brains of the flies [193]. Herbal medicines with a historical context in PD treatment, such as the squamosamide derivative FLZ, geldanamycin, celastrol, and arimoclomol, offer promising options due to active compounds with various anti-parkinsonian mechanisms of action [194,195,196,197]. FLZ is a novel synthetic derivative of squamosamide that is known for its ability to protect dopaminergic neurons from apoptosis [198,199]. Similar to the previously mentioned compound, FLZ has also been identified as an inducer of HSPs. In a study performed by Xiu-Qi Bao et al., the researchers demonstrated that treatment with FLZ in mice expressing mutant α-syn (A53T) improved motor function and dopaminergic nerve cell function. Moreover, FLZ alleviated neurotoxicity in SH-SY5Y cells overexpressing α-syn, leading to a reduction in α-syn expression and aggregation through HSP70. The neuroprotective effect of this compound is indeed attributed to the increase in the activity of HSP70, which occurs through to the direct binding of FLZ with HSP70-interacting protein (Hip), a co-chaperone of HSP70 [197].

In another study, andrographolide, a natural compound found in *A. paniculate*, mitigated MPTP toxicity in mice by inducing HSF1 via NRF2. Specifically, HSF1 has been suggested to mediate proteostasis by upregulating the activity of chaperones such as HSP70, as well as the CHIP-dependent pathway. Mice treated with andrographolide showed restored motor function, reduced α-syn levels, and increased HSP70 and HSF1 protein levels. This process was dependent on HSF1 and CHIP, as confirmed by specific siRNA knockdown of the HSF1 and CHIP genes in Neuro-2A cells treated with andrographolide [200].

The advantages of these natural compounds lie in their ability to induce HSP70 and HSP-related pathways, addressing multiple factors contributing to PD pathogenesis. However, these findings primarily come from preclinical models and further confirmation in alternative models is necessary to assess their translational potential for clinical studies.

#### 5.2.2. The Activation of HSP27 by Natural Compounds

Studies have shown that biological compounds derived from sea cucumbers can alleviate the neurotoxicity induced by α-syn aggregates, helping the recovery of dopaminergic neurodegeneration in *C. elegans* PD models by regulating the expression of HSPs [201,202]. For instance, decanoic acid extracted from the ethyl acetate fractions (HLEA-P1) of sea cucumber (*H. leucospilota*) has demonstrated the ability to alleviate dopaminergic neurodegeneration, reduce α-syn aggregation, and improve behavior in a *C. elegans* PD model. HLEA-P1 treatment induced the DAF-16 transcription factor target gene sod-3 and the HSP27 homologs HSP16.1 and HSP16.2, thereby improving resistance to oxidative and protein damage stress [201]. In a separate study, Chalorak et al. found that frondoside A, a natural triterpene glycoside from sea cucumber (*C. frondosa*), has the potential to promote the clearance of α-syn or prevent its accumulation by facilitating protein folding and degradation processes. Frondoside A achieves this reduction by enhancing the UPS pathway and increasing the expression of HSPs. Treatment with frondoside A extended the lifespan of *C. elegans* overexpressing α-syn compared with the control group. This extension in lifespan was linked to the upregulation of HSF1, UBH4, HSP16.1, and HSP16.2 [202]. Furthermore, HSP27 levels increase after food supplementation with amalaki rasayana in Drosophila models. Amalaki rasayana, derived from *Phyllanthus emblica* fruits, enhances cellular stress tolerance by reducing ROS production and lipid peroxidation, while also increasing SOD activity. Although the expression of HSP70 and HSP83 remains unaffected by amalaki rasayana supplementation, upregulation of HSP27 is especially notable in wild-type control flies and heat-shocked larvae. However, the protective effects of amalaki rasayana may depend on the presence of specific genes, such as parkin or Dj-1β. Mutant Drosophila models of PD, Park13 and DJ-1β^Δ93^, exhibit only partial or no protection against oxidative stress induced by paraquat [203]. These preclinical studies showcase the potential of sea-cucumber-derived compounds in mitigating PD-related neurodegeneration and α-syn aggregation through the regulation of HSPs. The advantages of these compounds lie in their ability to modulate critical cellular mechanisms. However, further investigations in alternative models are needed to validate these findings and explore their translational potential. These studies underscore the importance of continued research into novel therapeutic approaches for PD based on natural compounds and cellular stress responses.

#### 5.2.3. Impact of Natural Compounds on ER Stress

Various phytochemical compounds, including polyphenols and alkaloids, have been explored for their potential in PD treatment due to their neuroprotective, antioxidant, and anti-inflammatory properties [204]. Nicotine, the primary alkaloid in tobacco, has shown promise in preventing PD by reducing ER stress. It protects nerve cells by inhibiting the expression of GRP78 and CHOP induced by neurotoxic agents in both in vitro and in vivo models. The activation of the nAChRs-PI3K/Trx-1 pathway is associated with these neuroprotective effects [205].

Quercetin, a plant flavonol, has demonstrated neuroprotective effects by suppressing ER stress and cell death [206,207]. It prevents apoptosis and necrosis by reducing GRP78, CHOP, and p-eIF2α/eIF2α levels, particularly when dopaminergic neurons are exposed to neurotoxic agents. The study highlighted the protective role of quercetin by inhibiting CHOP [208]. Quercetin’s ability to suppress ER stress and protect dopaminergic neurons holds promise for potential therapeutic interventions in PD.

Resveratrol, a natural polyphenol, has neuroprotective potential due to its scavenging activity and its ability to activate stress response chaperones [209,210]. In a rat model of PD induced by 6-OHDA, resveratrol liposome treatment increased the phosphorylation levels of TRAP1 and PINK1, protecting against mitochondrial dysfunction [211]. TRAP1 plays a role in maintaining mitochondrial function, regulating apoptosis, and participating in the UPR in the ER. Inhibiting TRAP1 has been linked to neurotoxic effects [212]. However, after 2 weeks of resveratrol liposome treatment in rats induced with 6-OHDA, PINK1 levels and the phosphorylated TRAP1/TRAP1 ratio returned to normal. These findings, along with the improved mitochondrial activity and antioxidant effects, likely contribute to the survival of dopaminergic neurons in PD rats [211]. Additionally, resveratrol improved processes related to protein folding, degradation, and cellular redox balance in human skin fibroblasts from a PD patient with a parkin mutation. Most proteins with altered expression in PD were associated with ER protein processing, including GRP78 (HSPA5), HSP90B1, and HSPA8. Notably, the resveratrol treatment of PD cells upregulated HSPA8 and HSP90 levels, potentially promoting chaperone-mediated autophagy [213].

The traditional Chinese medicinal prescription, *Wuzi Yanzong prescription* may improve motor symptoms and reduce tissue damage in the substantia nigra of mice by affecting the UPS and preventing ER-stress-induced apoptosis. In this context, intraperitoneal injection of MPTP has been used as a PD model. Following treatment with *Wuzi Yanzong prescription*, there was a reduction in GRP78 levels in the striatum and cortex, as well as decreased levels of p-PERK, p-eIF2α, ATF4, p-IRE1α, XBP1, and CHOP [214].

Another commonly used Chinese herbal medicine, *Uncaria rhynchophylla*, has shown neuroprotective effects in PD models by suppressing HSP90 expression. This study highlighted that inhibiting HSP90 could ameliorate PD via the MAPK and PI3K-AKT signaling pathways. These results were further validated in a PD mouse model, where *Uncaria rhynchophylla* inhibited HSP90 expression, thereby ameliorating the dopaminergic neuron loss and behavioral deficits induced by MPTP exposure in mice [215]. Traditional Chinese medicines offer a holistic approach to PD treatment and their impact on multiple pathways suggests their potential as complementary therapies.

These studies demonstrate the potential of various phytochemicals in mitigating PD-related neurodegeneration, with advantages including their neuroprotective, antioxidant, and anti-inflammatory properties. However, it is essential to recognize that these findings are based on preclinical models, and further research is needed to confirm their efficacy in humans. Future investigations should focus on translational and clinical studies to determine the feasibility and safety of these approaches in PD patients. Furthermore, the combination of various phytochemicals or therapies may generate synergistic benefits, necessitating further investigation in future research.

**Table 2:** Therapeutic compounds as potential treatments for PD: insights from preclinical studies on stress protein modulation.

Treatment	Model	HSP Interaction	Effect	Ref.
Effects of commonly used compounds on HSP modulation in PD models				
Selegiline	Primary cultures of hippocampal-derived NSCs were pretreated with various concentrations of selegiline (0, 10, 20, 30, and 40 µM) for 48 h, followed by treatment with H_2_O_2_ (125 µM) for 30 min. The positive control cells were cultured for 48 h at 37 °C, then treated with H_2_O_2_ for 30 min.	HSPA4	Selegiline increased HSPA4 and Bcl-2 mRNA expressions, improving cell viability and reducing oxidative-stress-induced cell death.	[171]
Lapatinib	Wistar albino rats received subcutaneous injections of the vehicle, ROT (2 mg/kg), or lapatinib (100 mg/kg/day) orally administered 1 h after ROT injection. The experiment was conducted for a total of 21 days.	HSP90/CDC37 chaperone complex	Lapatinib improved motor deficits in ROT-induced rats and reduced nigrostriatal dopaminergic depletions. Reduction in HSP90, CDC37, and c-SRC levels resulted in neuroprotective effects in ROT-induced rats by suppressing the main proteins involved in PD pathogenesis (α-syn, LRRK2, and c-ABL).	[177]
Rifampicin	PC12 cells were differentiated with 50 ng/mL of nerve growth factor for 6 days and then treated with rifampicin at 150 μM for 24 h. Gene silencing was performed using GRP78-specific siRNA or control siRNAs for 24 h. Then, rifampicin (150 μM) was administered for 2 h, followed by 1 μM ROT for 24 h.	GRP78	Pretreatment with rifampicin provided neuroprotection by inducing GRP78 in a time- and dose-dependent manner. This upregulation occurred via the PERK-eIF2α-ATF4 pathway.	[180]
Empagliflozin	Wistar rats received 11 subcutaneous injections with the vehicle or ROT (1.5 mg/kg in 1% DMSO). The other animals were treated with empagliflozin alone (10 mg/kg/day orally) or empagliflozin with ROT starting from the 11th day, for 21 days.	GRP78	Empagliflozin improved histopathological alterations and motor deficit in ROT-induced rats by inhibiting GRP78/PERK/eIF4α/CHOP. Empagliflozin enhanced α-syn clearance by improving autophagy and UPS impairments.	[182]
Metformin	C57BL/6 mice received a stereotaxic injection of ROT (2 mg/kg), metformin (50 mg/kg), or metformin for 3 days followed by ROT treatment.	GRP78	Metformin pretreatment attenuated ER stress via inhibition of ATF4, ATF6, XBP1, GRP78, and CHOP mRNA levels, ameliorating dopaminergic neuron degeneration.	[183]
Metformin	Fibroblasts isolated from the skin biopsies of PD patients with R47X mutations or healthy patient controls and HeLa, SH-SY5Y, and HEK293 cells were cultured at suitable conditions. Metformin (10 mM) was used as a treatment for 24 h. Fibroblasts from TRAP1 or HTRA2 knockout mice were also used for the experiments. Other analysis required vectors containing TRAP1 and HTRA2 cDNA and specific siRNA for TRAP1, HTRA2, and controls.	TRAP1	R47X TRAP1 mutations could lead to upregulation in mitochondrial UPR and enhancement of mitochondrial membrane potential as a response to an imbalance of proteins, including HSP90, HSP60, and HSP70. Metformin was able to reverse the effect of mutation.	[185]
Sodium salicylate (HSF-1 inducer)	Sprague Dawley rats were treated with subcutaneous injection of vehicle, i.p. injection of ROT (2 mg/kg suspended), ROT + sodium salicylate (100 mg/kg; i.p. injection), or sodium salicylate alone for five weeks.	HSF-1, HSP-40, and HSP-27	Sodium salicylate protects the proteasome from oxidative stress and induces the expression of HSF-1, HSP-40, and HSP27, thereby also reducing α-syn aggregation.	[179]
Natural therapies modulating HSPs for neuroprotection in PD models				
Melatonin	SH-SY5Y cells were differentiated by using 10 μM retinoic acid for 6 days and subsequently exposed to melatonin (0.1, 1, 10, and 100 µM/mL) for 24 h. MPP^+^ (400 μM) was used to induce the PD model. For gene silencing, cells were transfected with HSF1-specific siRNA or control siRNAs for 24 h, then the cells were incubated with melatonin and MPP^+^ for 24 h.	HSF1 and MSP70	Melatonin increased HSF1 and HSP70 compared with the MPP^+^ group. HSF1 silencing resulted in HSP70 downregulation and lower protection, whereas pro-apoptotic proteins and oxidative stress were increased.	[188]
Glutamine	SH-SY5Y cells overexpressing α-syn were obtained using transfection with recombinant plasmid. Cultured cells were treated with glutamine (0, 2, 4, 8, or 16 mM) for 6, 12, 24, and 48 h. Gene silencing was performed using HSF1-specific siRNA or control siRNA.	HSF1 and HSP70	Glutamine increased both HSP70 mRNA and protein expression. This upregulation was dependent on HSF1 activation. This activation resulted in increased α-syn degradation.	[190]
Glutamine	PC12 cells overexpressing α-syn were achieved using transfection with recombinant plasmid. Cultured cells were treated with glutamine (0, 5, 10, or 20 mM) for 0, 4, 8, 12, 24, or 48 h. Gene silencing was performed using HSF1-specific siRNA or control siRNA.	HSF1 and HSP70	Glutamine increased both HSP70 mRNA and protein expression. This upregulation was dependent on HSF1 activation. This activation resulted in increased α-syn degradation.	[191]
Ethyl acetate fraction from *Holothuria leucospilota*	Transgenic BY250, NL5901, CF1553, CL2166, TJ356, and wild-type N2 strains were used as *C. elegans* models for the study. The worms were exposed to 6-OHDA followed by compounds isolated from the ethyl acetate fraction from sea cucumber (1, 5, 25, and 50 μg/mL). Compounds were mixed with *E. coli* OP50 and then administered to worms as a food source.	HSP27 homologs (HSP16.1, HSP16.2, and HSP12.6)	Decanoic acid from the ethyl acetate fraction at low doses protected dopaminergic neurons against α-syn aggregation and improved motor deficits in worms. These effects occurred via activating DAF16 and its downstream genes *sod-3, HSP16.1, HSP16.2*, and *HSP12.6*.	[201]
Frondoside A	Frondoside A was administered at doses of 0.1, 0.5, 1, 5, and 10 μM to *C. elegans* strains exposed to 50 mM 6-OHDA. BZ555 and NL5901 were used as mutant strains of *C. elegans*. N2 was employed as wild-type control strain.	HSF1, HSP27 homologs (HSP16.1, HPS16.2)	Frondoside A (1 µM) reduced α-syn aggregation by enhancing the UPS and HPSs expression.	[202]
Nicotine	PC12 cells were pretreated with nicotine (100 nM) or inhibitors (LY294002 and methyllycaconitine) 30 min prior to exposure to MPP+ treatment (0.3 mM). Then, cells were incubated for 24 h. For the PD animal model, C57BL/6 mice were i.p. injected with 20 mg/kg MPTP-HCl twice daily for 7 days. The nicotine (0.25 mg/kg twice daily for 7 days) was i.p. injected alone or 30 min prior MPTP administration.	GPR78 and CHOP	Nicotine enhanced cell viability and attenuated MPP^+^-induced neurotoxicity by reducing the GRP78 and CHOP ER stress proteins.	[205]
Sesamol and Naringerin	Male Wistar rats received i.p. injection of vehicle or ROT (3 mg/kg). Sesamol (15 mg/kg) and naringenin (10 mg/kg) was administered orally. Flavonoids were administered for 10 days after the 11 days of ROT treatment.	HSP70 and HSP90	Sesamol and narigerin promoted neuronal survival and improved muscle health by restoring protective proteins and increasing HSP70 and HSP90.	[192]
Quercitin	Dopaminergic SN4741 cells were pretreated with quercetin (10, 20, or 40 µM) 1 h prior the treatment with dieldrin (20 µM) for a total of 48 h. Gene silencing was performed using CHOP-specific siRNA or control siRNA.	GRP78, CHOP	Quercitin suppressed dieldrin-induced apoptosis in a dose-dependent manner, restoring CHOP and GRP78 levels and the p- eIF4α/eIF4α ratio induced by dieldrin.	[208]
Resveratrol liposomes (*Polygonum cuspidatum*)	The experiments were performed in Wistar rats through intracerebroventricular injection of 6-OHDA (15 μg) or vehicle or without injection. Resveratrol liposome was administered orally at a concentration of 20 mg/kg once daily for 2 weeks.	TRAP1 (HSP75)	Resveratrol liposome protected mitochondrial respiratory chain function and inhibited apoptosis in the substantia nigra of PD rats by increasing the phosphorylated TRAP1/TRAP ratio and PINK1 levels.	[211]
Resveratrol	Primary skin fibroblasts from a PD patient with a heterozygous parkin mutation and a healthy subject from the same family (control) were isolated from skin biopsy and cultured under suitable conditions. Then, the cells were treated with resveratrol (25 µM) or vehicle alone (0.2% DMSO). Proteomic analysis was performed using 2-DE, MALDI-TOF-MS, and Western blot.	HSP90B1, GPR78 (HSPA5), HSPA8	Resveratrol improved protein folding regulation by modulating chaperones levels, including HSPA8 and HSP90, as well as SIRT1 deacetylase, resulting in improved chaperone-mediated autophagy.	[213]
Spirulina/C-phycocyanin (Arthrospira platensis)	Dried spirulina powder was added in fly food media to obtain 5% and 10% *w*/*v* concentrations. Wild-type Oregon R+ and transgenic *DJ-1β^Δ93^* strains of Drosophila melanogaster were used as the control and PD models, respectively.	HSP70	Spirulina supplementation increased the survival of flies and improved antioxidant defenses against cellular stress by reducing the HSP70 and JNK signaling pathways.	[193]
*Amalaki rasayana*	Amalaki rasayana was mixed with fly food media to obtain a 0.5% *w*/*v* concentration. Wild-type Oregon R+ and the transgenic *DJ-1β^Δ93^* and *Park13* strains of *Drosophila melanogaster* were used as the control and PD models, respectively.	HSP27	*Amalaki rasayana* enhanced the tolerance to cellular stresses. This improvement was attributed to reduced ROS levels and lipid peroxidation, as well as an increase in SOD activity and HSP27 levels.	[203]
FLZ, a synthetic derivative of squamosamide (HSP70 inducer)	SH-SY5Y cells were transfected to produce a mutated α-syn (A53T) and then were exposed to 10 μM FLZ for 24 h. In the animal model, C3H mice expressing mutant α-syn received oral administration of 75 mg/kg FLZ for 7 weeks. However, gene silencing was performed using specific siRNAs for HSF1, HSP70, and co-chaperone genes.	HSP70 and its co-chaperone Hip	FLZ increased the expression of the co-chaperone Hip, enhancing HSP70 activity. FLZ directly bound to Hip and promoted its interaction with HSP70, which in turn reduced cytotoxic α-syn aggregates.	[197]
*Wuzi Yanzong* prescription	C57BL/6 mice were divided into a control group, a PD group (i.p. injection of MPTP for 1 week), and a PD + *Wuzi Yanzong* prescription group (16g/kg twice a day for 14 days).	GRP78, CHOP	*Wuzi Yanzong* prescription inhibited the UPR and ER-stress-induced apoptosis, possibly leading to an improvement in both PD symptoms and lesions. Specifically, the GRP78, p-PERK, p-eIF2α, ATF4, p-IRE1α, XBP1, ATF6, and CHOP ER related proteins were decreased after pretreatment in PD mice.	[214]
*Uncaria rhynchophylla* extract	Dried *Uncharia rhynchophylla* was used at concentrations 5, 10, or 20 µg/mL to treat SH-SY5Y cells for 6 h. Then, cells were exposed to MPP^+^ (1 mM) for 24 h. In the animal experiment, C57BL/6 mice were treated with vehicle, MPTP (30 mg/kg), or Uncharia rhynchophylla (20, 40, or 80 mg/kg orally) with or without MPTP. The experiment occurred for 19 days, with treatment from the 8th to the 12th day.	HSP90	*Uncaria rhynchophylla* extract enhanced cell viability in vitro by modulating apoptotic and autophagic pathways through the inhibition of HSP90 expression. Thus, the compound improved behavioral deficits and increased DA concentrations.	[215]
Andrographolide	Male Swiss albino mice were pretreated with MPTP (25 mg/kg), followed by andrographolide (10 mg/kg). MPTP (5 applications) and andrographolide (10 applications) were administered on alternate days for 20 days. The study included control mice and mice treated with andrographoline alone. HCT116, HEK293, and Neuro-2A cells were used for in vitro studies.	HSF1, HSP70	Andrographolide reduced the α-syn aggregation induced by MPTP via HSF1/HSP70, enhancing the protein quality control machinery. Additionally, andrographolide stimulated CHIP and ATG7 activity, increasing UPS activity and the autophagy process.	[200]

HSPs: heat shock proteins; PD: Parkinson’s disease; NSC: neural stem cell; H_2_O_2_: hydrogen peroxide; HSPA4: heat shock protein family A (Hsp70) member 4; Bcl-2: B-cell lymphoma 2; mRNA: messenger RNA; ROT: rotenone; DMSO: dimethylsulfoxide; CDC37: Hsp90 co-chaperone; α-syn: alpha synuclein; Cdc37; LRRK2: leucine-rich repeat kinase 2; GRP78: glucose regulated protein-78; siRNA: short-interfering RNA; PERK: protein kinase R (PKR)-like endoplasmic reticulum kinase; eIF2α: eukaryotic translation initiation factor 2A; ATF4: activating transcription factor 4; IREα: inositol-requiring transmembrane kinase endoribonuclease-1α; p-IREα: phosphor-inositol-requiring transmembrane kinase endoribonuclease-1α XBP1: x-box binding protein 1; ATF6: activating transcription factor 6; CHOP: C/EBP homologous protein; UPS: ubiquitin–proteasome system; i.p. intraperitoneal; HSF-1: heat shock factor-1; GSH: glutathione; MPP^+^: 1-methyl-4-phenylpyridine; sod-3: superoxide dismutase 3; *C. elegans*: Caenorhabditis elegans; 6-OHDA: 6-hydroxydopamine; MPTP: 1-methyl-4-phenyl-1,2,3,6-tetrahydropyridine; Trx-1: thioredoxin-1; ER: endoplasmic reticulum; p- eIF4α: phosphorylated eukaryotic initiation factor 4α; eIF4α: eukaryotic initiation factor 4α; TRAP1: TNF receptor associated protein 1 (heat shock protein 75 kDa); PINK1: PTEN-induced kinase 1; SIRT1: sirtuin 1; JNK: c-Jun N-terminal kinases; SOD: superoxide dismutase; FLZ: N-(2-(4-hydroxy-phenyl)-ethyl)-2-(2,5-dimethoxy-phenyl)-3-(3-methoxy-4-hydroxy-phenyl)-acrylamide; Hip: HSP70-interacting protein.

## 6. Challenges and Future Prospects

**Figure 5:**
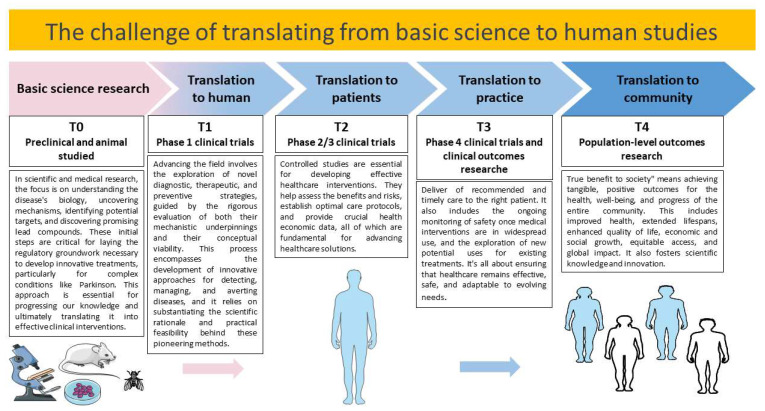
Translational research involves several operational phases (T0-T4) and the associated challenges that must be addressed. T0 represents basic science research focused on understanding cellular mechanisms, their links to diseases, and the identification of potential therapeutic targets, along with the development of new treatment methods, such as new drugs. T1 encompasses proof-of-concept studies conducted in human volunteers during phase 1 clinical trials. These studies aim to establish the safety, mechanisms, and feasibility of the proposed concept. T2 involves phase 2 and 3 clinical trials, ideally randomized, to test the effectiveness of the therapeutic agent in patient populations that represent the specific disease under consideration. These trials often include control groups for comparison. T3 is associated with phase 4 clinical trials, which aim to optimize the practical use of a therapeutic agent in clinical settings. T4 refers to population-level outcomes research or comparative effectiveness research, which assesses the long-term utility and cost-effectiveness of a therapeutic agent in comparison with other treatments in current use. Translating findings from basic science to human studies is a critical and challenging process. The image was created using the image bank of Servier Medical Art (available online: http://smart.servier.com/, accessed on 30 October 2023), licensed under a Creative Commons Attribution 3.0 Unported License (available online: https://creativecommons.org/licenses/by/3.0/, accessed on 30 October 2023).

The results of the studies described in this review highlight how the role of HSPs in PD is opening new perspectives in the field of the research and treatment of this condition. However, it is important to note that much of this research is still in the preclinical phase and requires further studies and trials to be translated into effective clinical therapies. Discoveries obtained from basic research are translated to the treatment and prevention of human diseases. It is important to understand intermediate steps such as the identification of targets, drugs, and biomarkers in animal models and human tissue xenotransplantation models. However, it appears difficult to translate these discoveries into clinical practice [216] (Figure 5).

The studies summarized in this review suggest that inducing or modulating HSPs, such as HSP70, HSP90, and others, could represent an important therapeutic strategy for PD. In the future, the development of drugs or gene therapies aimed at increasing the expression of specific HSPs or inhibiting HSP90 to protect nerve cells from death and reduce the accumulation of α-syn could be an interesting strategy. The identification of molecules capable of inducing HSP expression or inhibiting HSP90 is also a promising direction. These compounds could be developed as drugs for PD. Some of these HSP90 inhibitors have shown promising results in preventing α-syn aggregation and protecting nerve cells. On the other hand, targeted gene therapy to modulate the expression of specific HSPs, such as HSP70 or GRP78, could represent an innovative approach. This strategy could be used to enhance the brain cells’ ability to manage misfolded proteins and protect against cellular stress. Cellular therapies, such as stem cell transplantation, could also benefit from the use of HSPs. HSPs can help transplanted cells survive and integrate better into the brain environment, improving the effectiveness of these therapies.

The overview of the studies provided in this review also indicates that existing or new compounds modulating HSPs could be developed as pharmacological therapies for PD. For example, drugs such as selegiline, lapatinib ditosylate, empagliflozin, and others could be further examined in clinical studies for their effectiveness at slowing the progression of the disease or improving symptoms in PD patients. Therapies involving the modulation of HSPs could be used in combination with other standard treatments, such as dopaminergic therapy, to enhance the overall effectiveness of PD treatment. Studies on natural compounds such melatonin, glutamine, flavonoids, and other herbal derivatives may lead to the development of alternative or complementary therapies based on natural substances. The evaluation of beneficial effects in currently available animal models is limited because no preclinical model can faithfully replicate all the characteristics of PD pathology [217]. Research on the therapeutic compounds for PD reveals critical gaps. First, there is a need to translate promising preclinical findings into practical applications in human trials. Many preclinical studies are of relatively short duration, which poses challenges for the accuracy and reliability of clinical trials. Extended-duration studies are required to fully understand the long-term safety and efficacy of therapeutic compounds that target stress proteins in PD [218].

Toxin-based models are adequate for studying the effects of dopamine replacement therapies but may not adequately mimic the slow, progressive neurodegeneration observed in PD patients. Even in genetic models, the level of nigrostriatal pathology is relatively modest in most models and a strong disease phenotype is clearly lacking [219]. An approach combining genetic models with the application of neurotoxins may provide a new concept that is more suitable for the preclinical testing of neuroprotective strategies [220]. Different animal models have been produced for studying PD, using neurotoxic chemicals, such as 6-OHDA, MPTP, or ROT, or genetic modifications. In both cases, the methodology has advantages and disadvantages; however, currently there is no perfect PD model for research [221]. For example, the function of the pro-apoptotic protein CHOP has been observed to depend on the particular toxic stimulus. Specifically, the lack of CHOP due to a null mutation resulted in neuroprotective effects in animals exposed to 6-OHDA but not in animals treated with MPTP, even though CHOP was expressed in both models [62].

The design of clinical trials to assess neuroprotective effects in PD has been a complex challenge. Patient selection, intervention timing, target determination, and biomarker data acquisition are significant hurdles. PD exhibits heterogeneity in terms of etiology and symptoms, making it difficult to prevent the cellular damage caused by misfolded protein accumulation [222]. Enhancing the function of HSPs holds promise in preventing misfolding, accumulation, and protein aggregation, thereby averting cell death. Nevertheless, it remains to be understood whether a single targeted treatment is adequate, given the involvement of various pathological pathways. A combination of treatments that stimulate molecular chaperones and facilitate protein clearance may represent a promising approach.

Notably, research on HSPs may lead to the identification of useful biomarkers for early PD diagnosis and for assessing the effectiveness of treatments. These biomarkers could be used for early diagnosis, monitoring disease progression, and evaluating treatment effectiveness. Despite significant efforts, it is notoriously difficult to establish reliable biomarkers for the preclinical phase of PD. To translate these results into clinical practice, rigorous clinical trials are required. Some studies are already underway to assess the therapeutic potential of certain HSP-related molecules in PD. These clinical trials will be crucial in determining the effectiveness and safety of HSP-based treatments.

The studies may lead to the identification of PD patient subgroups that will benefit more from HSP modulation. Future therapies could be personalized based on the genetic and biomolecular profiles of patients, allowing treatment to be more precisely tailored to the specific needs of each individual patient.

## 7. Conclusions

Several therapeutic interventions for PD have highlighted that modulating HSP expression may result in neuroprotective effects. The inhibition of HSP90 may enhance the induction of protective HSPs, including HSP70 and HSP27. Upregulating HSP70 and HSP27 has been observed to promote α-syn degradation, thus mitigating dopaminergic neurodegeneration. However, gene therapy has emerged as a potential strategy to overcome the drug’s toxicity and limited penetration into the blood–brain barrier. Several pharmacological and natural compounds regulate pathological changes in PD by targeting specific chaperone proteins and reducing ER stress, including the GRP78/CHOP pathway. This may improve cell survival, ultimately ameliorating PD pathology and symptoms. However, these studies are preclinical; thus, clinical studies will be needed to confirm the efficacy and safety of these treatments in PD patients.

## Figures and Tables

**Table 1:** Overview of preclinical studies describing how several strategies and/or therapeutic molecules exert a neuroprotective effect in PD by modulating stress proteins.

Treatment	Model	HSP Interaction	Effect	Ref.
HSP inducers				
HSP70 exogenous	SH-SY5Y cells were exposed to ROT (3 μg/mL) for 1 h to induce the PD model and then treated with HSP70 (5, 10, 15, or 20 mg/L) for 72 h.	HSP70	HSP70 reduced ROS and lipid peroxidation levels and protected cells from the apoptosis and mitochondrial dysfunction ROT-induced. Additionally, it reduced the levels of the NF-κB and STAT3 proteins.	[91]
HSP70 exogenous	Astrocytes were transfected with HSP70 cDNA and 4 h later were exposed to 100 nM of α-syn protein (A53T, human). For Hsp70 inhibition, 5 h before α-syn exposure, cells were exposed to VER155008 (10 μM).	HSP70	HSP70 overexpression attenuated the neuroinflammatory and neurodegenerative response through inhibition of the JNK and NF-κB signaling pathways.	[92]
HSP70 exogenous	Male Wistar rats were subjected to several repeated bilateral microinjections of lactacystin (4 μg/μL) in the substantia nigra pars compacta every 7 days to induce PD. Then, 4 and 24 h after each lactacystin microinjection and a week after the last, the animals were treated the recombinant inducible human HSP70i (5 μg/10 μL) into each nostril of the rats.	HSP70	HSP70i reduced the loss of dopaminergic neurons in the substantia nigra pars compacta that lactacystin induced, improved behavioral parameters in animals, and restored tyrosine hydroxylase levels.	[93]
HSP70 overexpression	Different fly strains were used, such as the control strain (w1118), the strain that did not express HSP70 (Df Hsp70), a dominant negative mutant strain of HSP70 (Hsp70K71E), a strain that overexpressed HSP70 (UAS-Hsp70), and a strain that overexpressed the human version of HSP70 (HSPA1L). All these mutants were exposed to paraquat (10 or 20 mM) for 12 and 24 h in order to induce PD-like symptoms.	HSP70	The HSP70 overexpression in Drosophila mutants protected dopaminergic neuronal cells from oxidative stress and avoided the neuronal death that parquet induced. HSP70 also improved locomotor deficits in Drosophila and ameliorated survival.	[94]
Echinochrome derivative U-133 (HSP70 inducer)	A lactacystin-induced rodent PD model was treated with injections (i.p.) of echinochrome derivative U-133 at a dose of 5mg/kg 4 h and 24 h after each microinjection of irreversible proteasome inhibitor lactacystin and 7 days after.	HSP70	HSP70 overexpression, via the U-133 HSP70 inducer, in dopaminergic neurons of the substantia nigra pars compacta protected the animals from lactacystin-induced PD-like symptoms.	[97]
Carbenoxolone (HSF-1 inducer)	The ROT model of PD was used for the study. The animals were divided into a control, ROT (ROT suspended in sunflower oil at a dose of 2 mg/kg; i.p.), ROT (ROT suspended in sunflower oil at a dose of 2 mg/kg; i.p.) + carbenoxolone, and carbenoxolone only (20 mg/kg; i.p.) groups for five weeks.	HSF-1 and HSP27	Simultaneous treatment of carbenoxolone and ROT for five weeks slowed the neurodegenerative process and improved motor functions in the rat PD model via the elevation of HSF-1 and HSP-27 expression.	[100]
CSPα phosphorylation	For the in vivo study, PKCγ-KO mice and control mice (mice with the *PKCγ* gene intact) were used. To study the effect of phosphorylation on CSPα, the researchers would introduce CSPα mutants, such as CSPα(S10A/S34A) and CSPα(S10D/S34D), into cultured cells to evaluate how they affected function and interaction with other proteins such as HSP70 and SNAP25.	PKCγ–CSPα–HSC70/HSP70–SNAP25 axis	CSPα phosphorylation by PKCγ can protect the presynaptic terminal from neurodegeneration.	[102]
CHEC-9 peptide (HSF-1 inducer)	Sprague Dawley rats were fed a diluted strawberry gelatin solution containing the CHEC-9 peptide or not (1.0 mg/kg). For the in vitro study, human SY5Y neural cells were previously exposed to ROT at a concentration of 0.04 μM. After 10 min, a substance called CHEC-9 or a vehicle (a substance with no effects) was added to the cells for 24 h.	HSP70	Oral treatment with the CHEC-9 peptide increased the level of active HSP70 monomers. In fact, it has been demonstrated in vitro that CHEC-9 also binds to HSP70 in the cytosol of the cerebral cortex. In the in vitro model of α-syn aggregation, CHEC-9 treatment induces HSP70-dependent dissolution of these aggregates in an HSP70-dependent manner.	[105]
Apelin-13	SH-SY5Y cells were treated with MPP+ (0, 100, 250, 500, 750, or 1000 μM) with or without apelin-13 (100 nM) for 36 h.	GRP78	Pretreatment with apelin-13 reduced ER stress through the inhibition of GRP78/CHOP/cleaved caspase-12 activation and the upregulation of phospho-ERK1/2, consequently reducing MPP+-induced apoptotic processes.	[110]
Apelin-13	C57BL/6 male mice were treated with apelin-13 (0.3 μg/mice/day) or the same volume of saline into the substantia nigra pars compacta for 12 days and then were administered with MPTP (25 mg/kg/day) or saline intraperitoneally for 5 days.	GRP78, CHOP, and XBP1	Apelin-13 exerted neuroprotective functions and improved motor impairments by inhibiting ER stress proteins and enhancing autophagy. It reduced levels of IRE1α, XBP1s, CHOP, and GRP78, which are stress proteins induced by MPTP. Furthermore, it decreased α-syn expression.	[111]
Apelin-36	In order to induce the PD model, C57BL/6 mice were injected intraperitoneally with MPTP (25 mg/kg/day) or saline for 5 days. Afterwards, the animals were treated with apelin-36 (0.5 μg/mice/day) or the same volume of saline injected into the substantia nigra pars compacta for 7 days.	GRP78 and CHOP	Apelin-36 attenuated MPTP/MPP+-induced neurotoxicity in vitro and in vivo by inhibiting ER stress, apoptosis, and α-syn expression. It reduced levels of GRP78, CHOP, and cleaved caspase-12 in MPTP/MPP+-treated mice and cells. Thus, apelin-36 improved motor dysfunction and dopaminergic neurodegeneration.	[112]
miR-384-5p	SH-SY5Y cells were transfected with miR-384-5p mimics or inhibitors (50 nM) for 24 h prior to ROT (20 μM) exposure. For gene silencing, cells were transfected with GRP78 siRNA (50 nM) or control siRNA was transfected into SH-SY5Y cells with or without miR-384-5p inhibitors.	GRP78	miR-384-5p inhibitors, inducing GRP78 overexpression, reduced α-syn-induced neurotoxicity by downregulating ER stress.	[115]
MANF	SH-SY5Y cells were cultured under different conditions: control, 6-ODHA (150 μM), 6-OHDA (150 μM) + MANF (4 μg/mL). Gene silencing was achieved using shRNA designed for the HSP70 target sequence (from 3476 to 3494 cDNA).	GRP78 and HSP70	MANF treatment led to ER stress gene overexpression, such as HSP70 and GRP78. HSP70 silencing suppressed MANF’s protective effect against 6-OHDA-induced cell death.	[118]
MANF	The experiment was performed on SH-SY5Y cells treated with different concentrations of 6-OHDA (0–125 μM) and MANF (4 µg/mL or 8 µg/mL) for 48 h. The overexpression of α-syn was obtained through plasmid transfection into cells. Additionally, GRP78 knockdown was carried out via transfecting a vector containing a shRNA specific for a sequence of GRP78.	GRP78	MANF suppressed apoptosis via GRP78 upregulation. The expression of GRP78 was related to cell survival, as demonstrated by using knockdown cells for GPR78.	[119]
CDNF and GDNF	Male Wistar rats received a unilateral stereotaxic injection of 6-OHDA (20 µg) in the left striatum. After 4 weeks, animals received unilateral intrastriatal injections of CDNF (1, 2.5, 5 μg) and GDNF (1, 2.5, 5 μg) alone or in combination. Primary cultured neurons from mouse embryos were exposed to thapsigargin (200 nM) and then to CDNF (100 ng/mL) and GDNF (50 ng/mL).	GRP78 and HSP70	CDNF + GDNF activated the PI3K-Akt pathway, promoting cell survival. However, only CDNF reduced the expression of ER stress markers, including ATF6, GRP78, and phosphorylation of eIF2α.	[121]
HSP90 Inhibitors				
SNX-0723 and SNX-9114 (Small molecule Hsp90 inhibitors)	Rats were injected unilaterally in the substantia nigra with AAV8 expressing human α-synuclein in order to induce a model of OD. Then, they were treated with SNX-0723 (10 mg/kg) and SNX-9114 (1.5 and 3 mg/kg) for 8 weeks by oral gavage.	HSP90 and HSP70	SNX-0723 (PF-04924868) and SNX-9114 (PF-04944733) protected against α-syn-dependent nigrostriatal toxicity through inhibition of Hsp90 and via upregulation of HSP70.	[125]
HSP90 inhibition (HSP90 siRNA)	Exposure of PC12 cells to 6-OHDA (75, 100, 125, 150, 175, and 200 μM) for 24 h and subsequent transfection with HSP90 siRNA (30 nM).	HSP90, HSF-1 and HSP70	Inhibition of HSP90 protects cells from death, reducing the expression of pro-apoptotic factors and conversely increasing the expression of antiapoptotic factors. Furthermore, suppression of HSP90 mediated the regulation of other proteins, including the transcription factor HSF-1, which is involved in the activation of protective genes against stress, such as HSP70.	[126]
Trichostatin A (a potent inhibitory of HDAC activity)	The UPS impairment model of PD, established by stereotaxic injection of lactacystin (1.25 μg/2 μL) or its vehicle into the right medial forebrain bundle of the mouse (C57BL/6). For treatment, mice received an i.p. injection of trichostatin A (2 mg/kg) or its vehicle every other day. For the in vitro study, SK-N-SH cells and primary ventral midbrain neuron cultures were treated with lactacystin (10 μM) or tubacin, niltubacin (specific HDAC6 inhibitors; 10 μM), and/or HSP90 inhibitors (5 or 10 μM).	HDAC6, HSP90, HSF1, HSP70, and HSP27	HDAC6 reduced α-syn oligomer levels and ameliorated the dopaminergic neuron survival in the UPS-impairment-induced PD model. This effect was due to HDAC6 binding to α-syn, which led to the triggering of chaperone expression, including HSP70 and HSP27, through dissociation of the HSP90–HSF1 complex.	[130]
Tubastatin A (selective inhibitor of HDAC6 activity)	Primary cultures of rat brain oligodendrocytes were incubated with MG-132 (1 μM) alone for 16 h or preincubated with tubastatin A (10 μM) for 3 h followed by MG-132 or for a further 16 h. The plasmid with shRNA for HDAC6 was transiently transfected into OLN-t40 cells (oligodendroglial cell line OLN-93 stably transfected with the longest human tau isoform) and after 24 h the cells were treated with MG-132 for 24 h.	HDAC6 and HSP70	Tubastatin-A-induced HDAC6 inhibition does not prevent the accumulation of protein aggregates induced by UPS inhibition by MG-132. It caused an altered stress response by repressing the activity of HSP70 and induced the accumulation of autophagosomal vacuoles.	[131]
Tubastatin A (selective inhibitor of HDAC6 activity)	A rat model was used in which PD-like neurodegeneration was induced via overexpression of human α-syn in the substantia nigra pars compacta. The HADC6 inhibition was induced by treatment of rats with 15 mg/kg tubastatin A daily by intraperitoneal injection for 12 days, two days after the PD model induction.	HDAC6 and the chaperone proteins HSC70 and Lamp2	HDAC6 could be potential as a therapeutic target, since inhibiting its activity via tubastatin A protected dopaminergic neurons against α-syn toxicity within the substantia nigra of rats. This effect was associated with an increase in chaperone-mediated autophagy via HSC70 and LAMP2.	[132]
Gene therapy				
Overexpression of HSP70	A SH-SY5Y cell line stably expressing human Hsp70 was used. For treatment, these cells were treated with proteasome inhibitor PSI (30 nM), 17-AAG (5 μM), and HSP inhibitor I (KNK-473, 20 μM). To assess the PD model, parkin-null flies *24B -Gal4* (muscle-specific) and *UAS-Hsp70* (*UASHsap/HSPA1L)*.W Bonini) were used. After eclosion, flies were treated with 17-AAG (5 μM) for 25 days.	HSP70	HSP70 induction protected cells against proteasome dysfunction. Treatment with 17-AAG, an HSP70 inducer, ameliorated the pathological phenotypes of Drosophila Parkin-null mutants	[134]
Overexpression of CHIP	In the animal model of Drosophila with genetic mutations in the PINK1 and PARK genes and wild-type (w1118), the overexpression of the CHIP protein was obtained by genetic transformation via a procedure called “p-element-mediated transformation”. To create flies without the CHIP protein (CHIP null mutant flies), a genome editing technique called CRISPR-Cas9 was employed.	CHIP	Overexpression of Drosophila CHIP suppressed the abnormal phenotypes and mitochondrial dysfunction in PINK1- or PARK-deficient flies thanks to its E3 ubiquitin ligase activity.	[136]
Overexpression of CHIP	SH-SY5Y cells were treated with 0.5, 1, 2, and 3 mM MPP+ for 24 h and then transfected with a CHIP-WT plasmid to overexpress human CHIP. Alternatively, cells were transfected with CHIP shRNA and a control shRNA to obtain CHIP knockdown and control cells, respectively. C57BL/6 mice were treated with MPTP to serve as a PD model. The overexpression of the CHIP protein was obtained via intravenous injection with AAV/BBB, whereas CRISPR/CAS9 was used to generate CHIP with a floxed STOP codon in the neural tissue of mice.	CHIP	Overexpression of CHIP reduced MPTP-induced toxicity, improving motor deficits and dopaminergic neuron survival. Moreover, CHIP overexpression suppressed the PD pathological upregulation of Drp1, proving the role of CHIP in mitochondrial maintenance.	[139]
AVV-CHIP	The delivery of AAVs-α-syn was employed to induce α-syn aggregates into the substantia nigra pars compacta of rats. Additionally, a separate vector containing CHIP cDNA was used to overexpress.	CHIP	CHIP overexpression reduced α-syn aggregates but may also affect tyrosine hydroxylase.	[140]
AVV-HSP70 and AVV-HSP27	Male Sprague Dawley rats were injected intranigrally with pathogenic AAV-α-syn, to induce the PD model and simultaneously with AAV-HSP27 or AAV-HSP70 viral vectors.	HSP70 and HSP27	AVV-HSP70 significantly reduced the dystrophy of nigrostriatal dopaminergic neurons and reduced the accumulation of α-syn in the substantia nigra. AVV-HSP27 did not protect animals from α-syn-induced pathology.	[145]
Recombinant HSP70	Male Wistar rats received an intrastriatal injection of 6-OHDA (20 μg/rat) or vehicle. After 5 days, each rat was treated with a daily intranasal dose of recombinant HSP70 (2 μg/rat) or saline for 15 days.	HSP70	Recombinant HSP70 counteracted the 6-OHDA-induced neurotoxicity, exerting anti-inflammatory effects, protecting dopaminergic neurons from death, and improving locomotor activity in animals.	[146]
GRP78 siRNA recombinant AAV	Male Sprague Dawley rats were injected into the substantia nigra pars compacta with pathogenic recombinant AAV-α-syn (1.5 μL) to induce the PD model and simultaneously with GRP78 siRNA recombinant AAV (1.5 μL).	GRP78	Reducing GRP78 levels in mouse brain cells worsened α-syn-induced neurotoxicity, and the severity increased with reduced protein expression.	[147]
AAV6 DNAJB6	In vitro, the HEK293 cell line was transfected with α-syn fibrils to induce α-syn aggregation. In vivo, stereotaxic injections of AAV6 vector cocktails (AAV-human wild type α-syn and AAV6-GFP or AAV6-human wild type α-syn and AAV6 DNAJB6-GFP) were performed into the substantia nigra of Sprague Dawley rats.	DNAJB6	DNAJB6 counteracted α-syn aggregation in vitro. In vivo, DNAJB6 prevented α-syn aggregation and this resulted in a decrease in dopaminergic cell death and PD-related motor deficits in an animal model of PD.	[148]
AAV6CSPα	PC12 cells stably expressing 1-120hαSyn were transduced with AAV6CSPα (AAV6 vector encoding human CSPα) or with an empty AAV6EV (AAV6 control vector). In vivo, 10-month-old 1-120hαSyn transgenic mice showing a progressive decrease in striatal dopamine release were injected bilaterally with 2 μL of AAVCSPα and AAVEV into the substantia nigra pars compacta.	CSPα	CSPα rescues an α-synuclein-aggregation-related phenotype in 1-120hαSyn mice. Viral CSPα administration ameliorated impaired synaptic function, reduced synaptic α-syn aggregations, and restored normal dopamine release in 1-120hαSyn mice. This also led to the restoration of normal dopamine release in the mice.	[152]
Hsp104 mutants	The dopamine transporter gene (dat-1) promoter was used to target the expression of Hsp104 mutants (Hsp104A437W or Hsp104A503V or Hsp104Y507C) and α-syn in *C. elegans* dopaminergic neurons.	HSP104	Enhanced HSP104 mutants improve aggregate dissolution, restore proper protein localization, suppress proteo-toxicity, and attenuate dopaminergic neurodegeneration.	[154]
Stem cell treatments				
hBM-MSC secretome	Five weeks after 6-OHDA induction, animals received the hBM-MSC secretome and levodopa. The animals received the hBM-MSC secretome by intracranial injection directly into the substantia nigra and striatum, whereas levodopa (12 mg/kg) was administered via oral gavage.	HSP27	The multifactorial protein composition of the secretome includes several factors, such as HSP27, that could be important in the neuroprotective and functional recovery effects.	[160]
hBM-MSCs secretome and hBM-MSCs transplants	Five weeks after 6-OHDA induction, animals received the hBM-MSC secretome and hBM-MSC transplants (200,000 cells in the substantia nigra pars compacta and in the striatum). In vitro, neural progenitor cells were exposed for 5 days to the hBM-MSC secretome.	UPS	The secretome showed a better effect in restoring dopaminergic neurons and improving motor functions compared with hBM-MSC transplantation.	[162]
BDNF-modified hUC-MSCs derived Dopaminergic-like neurons	hUC-MSCs were cultured and differentiated into dopaminergic-like neurons with a neural induction solution for 24 h. After 9 days, cells were supplemented with 50 ng/mL BDNF for 3 days. Lentiviral vectors carrying GFP-BDNF, GFP-null, or BDNF-siRNA were transfected into dopaminergic-like neurons. For cell transplantation, rats were treated with hUC-MSC dopaminergic-like neurons transfected with null vector, with BDNF vector, or with BDNF siRNA.	HSP60	The BDNF-modified hUC-MSC-derived dopaminergic-like neuron transplantation improved motor deficits, promoted neuroprotection and anti-inflammatory activity, and increased neuronal markers, intracerebral dopamine levels, and expression of proteins such as HSP60.	[165]

HSPs: heat shock proteins; PD: Parkinson’s disease; i.p. intraperitoneally; Hip: HSP70-interacting protein; NF-κB: nuclear factor kappa B; STA3: signal transducer and activator of transcription 3; JNK: c-Jun N-terminal kinase; hBM-MSC: human bone-marrow-derived mesenchymal stem cells; 6-OHDA: 6-hydroxydopamine; UPS: ubiquitin–proteasome system; HDAC6: histone deacetylase 6; α-syn: alpha synuclein; CSPα: cysteine (Cys) string protein α; PKC: protein kinase C; SNAP25: synaptosomal-associated protein 25; ROT: rotenone; HSF-1: heat shock factor-1; GSH: glutathione; MPP+: 1-methyl-4-phenylpyridine; GRP78: glucose-regulated protein 78; MANF: mesencephalic astrocyte-derived neurotrophic factor; CDNF: cerebral dopamine neurotrophic factor; GDNF: glial cell line-derived neurotrophic factor; PI3K: phosphoinositide 3 kinase; AKT: serine-threonine kinase; eIF2α: eukaryotic initiation factor 2α subunit; CHOP: C/EBP homologous protein; ERK 1/2: extracellular signal-regulated kinase 1/2; siRNA: small interfering RNA; shRNA: small hairpin RNA; MG-132: carbobenzoxy-L-leucyl-L-leucyl-L-leucinal; AAV: adeno-associated virus; CHIP: carboxyl terminus of Hsc70-interacting protein; PINK1: phosphatase and tensin homolog-induced kinase 1; PARK: parkin; CRISPR: clustered regularly interspaced short palindromic repeat; BDNF: brain-derived neurotrophic factor; hUC-MSCs: human umbilical cord mesenchymal stem cells; GFP: green fluorescent protein; dat-1: dopamine transporter gene.

## Data Availability

Not applicable.
